# Comparative effectiveness of exercise types and doses for improving pain intensity in women with fibromyalgia syndrome: a systematic review and dose–response meta-analysis

**DOI:** 10.3389/fphys.2026.1971006

**Published:** 2026-10-28

**Authors:** Xiangjie Sheng, Jin Cai, Xiaolong Mou, Peng Gao, Qiaonan Chen

**Affiliations:** 1 Department of Physical Education, Dongshin University, Naju, Jeollanam-do, Republic of Korea; 2 Department of Sports, Shandong University of Engineering and Vocational Technology, Jinan, Shandong, China; 3 Department of Sports, Rizhao Polytechnic University, Rizhao, China; 4 Shandong Vocational University of Foreign Affairs, Weihai, Shandong, China

**Keywords:** exercise, fibromyalgia syndrome, pain, systematic review, women

## Abstract

**Background:**

Fibromyalgia syndrome (FMS) is a chronic condition characterized by widespread pain and substantial impairments in physical and psychosocial functioning, predominantly affecting women. Although exercise is recommended as a non-pharmacological approach for pain management in FMS, the dose–response relationships between different exercise modalities and pain remain unclear. This study aimed to compare the associations of different exercise modalities and doses with pain intensity in women with FMS.

**Methods:**

Exercise dose was calculated as the product of duration, frequency, and intensity, expressed in metabolic equivalents of task minutes per week (MET-min/week), to enable comparisons across studies. Bayesian network and dose–response meta-analyses were conducted to compare the effects of different exercise modalities on pain and characterize their dose–response relationships. Pain was assessed using the Visual Analog Scale (VAS).

**Results:**

A total of 24 studies were included in the systematic review, involving 1992 women with FMS. Overall exercise demonstrated a positive dose–response association with pain improvement, with the greatest effect observed at approximately 1400 MET-min/week (MD = −3.02, 95% CrI: −5.42, −0.52). Stratified analyses showed that aerobic exercise (AE), resistance training (RT), and mind–body exercise (MBE) were significantly associated with pain improvement, although the effective dose ranges and doses associated with the maximum effects varied across modalities. The effective dose ranges were 290–1400 MET-min/week for AE, 400–550 MET-min/week for RT, and 140–800 MET-min/week for MBE. The greatest improvements were observed at 1400 MET-min/week for AE (MD = −2.93, 95% CrI: −5.36, −0.49), 550 MET-min/week for RT (MD = −1.81, 95% CrI: −3.60, −0.06), and 800 MET-min/week for MBE (MD = −3.07, 95% CrI: −4.99, −1.09). Meta-regression analyses found no significant moderating effects of the examined study-level characteristics.

**Conclusions:**

Different exercise modalities showed distinct dose–response relationships for pain improvement in women with FMS. MBE at approximately 800 MET-min/week was associated with greater improvement in pain intensity, although further high-quality randomized controlled trials are needed to confirm these findings.

**Systematic review registration**: https://www.crd.york.ac.uk/PROSPERO/view/CRD420261478256, identifier CRD420261478256.

## 1 Introduction

Fibromyalgia syndrome (FMS) is a chronic, multisystem condition characterized by widespread musculoskeletal pain, persistent fatigue, sleep disturbances, and impaired physical functioning (Marques et al., 2017; Sarzi-Puttini et al., 2020). It affects approximately 2–4% of the global population and disproportionately affects women (Cabo-Meseguer et al., 2017). Given its widespread and persistent symptoms, particularly chronic pain, FMS can substantially impair patients’ quality of life, work capacity, and social functioning (Annemans et al., 2009; Sarzi-Puttini et al., 2020).

Given the limited efficacy and potential adverse effects of pharmacological therapies, non-pharmacological approaches have become a cornerstone of FMS management (Wilson et al., 2012; Siracusa et al., 2021). Among these approaches, exercise interventions have gained increasing recognition as a low-risk and effective strategy for addressing the challenges posed by FMS (Mannerkorpi, 2005). The physiological mechanisms underlying these benefits may involve enhanced central pain modulation, improved cardiovascular function, and attenuation of systemic inflammation (Carvalho et al., 2020).

In recent years, several systematic reviews have investigated the effects of individual exercise modalities or compared different types of exercise in women with FMS, **c**onsistently demonstrating beneficial effects across various exercise modalities (Wang et al., 2025; Casanova-Rodríguez et al., 2026; Gao et al., 2026). Despite these findings, existing evidence has yet to address an important gap in current guidelines, namely, the lack of specific recommendations regarding the effective dose range and dose–response relationships of different exercise modalities for pain management in women with FMS. This lack of concrete exercise prescription parameters limits the clinical applicability of existing evidence. Exercise dose, including intensity, duration, and frequency, is a crucial component of exercise prescription. Given the vulnerability of individuals with FMS, inappropriate or excessive exercise doses may be ineffective or potentially exacerbate pain (Lima et al., 2017; Sluka et al., 2018). Although recent studies have explored the role of exercise dose in FMS, several limitations remain. Núñez et al. focused exclusively on aerobic exercise, leaving the dose–response relationships of other exercise modalities insufficiently investigated (Núñez-Cortés et al., 2025). Wang et al. examined the overall dose–response association of exercise; however, they did not model dose–response relationships separately for different exercise modalities and included mixed populations beyond women with FMS, which may introduce additional heterogeneity (Wang et al., 2025). Rodríguez-Domínguez and Niu et al. considered weekly exercise dose by dichotomizing exercise frequency, duration, or adherence (Niu et al., 2024; Rodríguez-Domínguez et al., 2025),, but this approach may overlook the continuous nature of exercise exposure and limit the ability to characterize dose–response patterns. Therefore, identifying effective doses across different exercise modalities and estimating dose-related benefits remain important for informing individualized exercise recommendations for women with FMS.

To fill this evidence gap, a Bayesian dose–response network meta-analysis provides a suitable analytical framework, as it enables the simultaneous evaluation of multiple exercise modalities and doses while accounting for between-study heterogeneity (Mawdsley et al., 2016). This approach offers two important advantages: (1) it allows the identification of effective doses for individual exercise modalities; and (2) beyond conventional network meta-analysis, which primarily focuses on comparing intervention effects, it enables the integrated assessment and ranking of different exercise modalities across dose levels, thereby providing more clinically informative evidence for exercise prescription. Therefore, this study aimed to compare the dose–response relationships between different exercise modalities and pain intensity in women with FMS based on evidence from randomized controlled trials (RCTs). The findings may provide quantitative evidence to support the development of individualized, dose-specific exercise strategies for pain management in women with FMS.

## 2 Methods

### 2.1 Protocol and registration

This systematic review and network meta-analysis was prospectively registered in PROSPERO (registration number: CRD420261478256) and performed following the PRISMA-NMA reporting guidelines (Hutton et al., 2015).

### 2.2 Search strategy

PubMed, Web of Science, Embase, and the Cochrane Central Register of Controlled Trials were searched systematically from database inception through July 12, 2026, with no restrictions on language. Search terms consisted of both free-text words and MeSH terms covering the target population (fibromyalgia syndrome), intervention (exercise), and study design (randomized controlled trials), with the concepts combined using the Boolean operator “AND.” The reference lists of relevant articles were also examined manually to locate additional eligible studies not captured by the electronic searches. Detailed search strategies for each database are provided in **Supplementary Content 1**.

### 2.3 Eligibility criteria

Eligibility criteria were established using the PICOS format (Population, Intervention, Comparison, Outcomes, Study Design) to minimize clinical bias in study selection (Tacconelli, 2010). **Table 1** summarizes the eligibility criteria for included studies based on the PICOS framework.

**Table 1:** Eligibility criteria formulated based on the PICOS framework.

PICOS	Inclusion criteria	Exclusion criteria
Population	Studies involving adult women aged ≥18 years with fibromyalgia syndrome, regardless of disease severity	Studies involving participants with major comorbidities, such as cardiovascular disease, cancer, or severe neurological disorders, were excluded to enhance population homogeneity
Intervention	Structured exercise (e.g., aquatic Exercise aerobic exercise, resistance exercise, mind–body exercise, or combinations of these modalities) interventions with clearly reported characteristics, including exercise type, frequency, duration, and session length, were eligible for inclusion to enable evaluation of exercise dose effects	Studies involving exercise interventions combined with other co-interventions (e.g., nutritional supplementation or dietary modification) were excluded unless the same co-intervention was provided to both the intervention and control groups. This criterion was applied to ensure that the observed effects could be primarily attributed to the exercise intervention rather than to concurrent interventions. Studies with incomplete or unclear information on exercise dose were also excluded to ensure the reliability and interpretability of the dose–response analyses
Comparison	A control group (e.g., usual care) was required. For direct comparison studies, the control group could include any form of exercise intervention, including different intensities within the same exercise modality	Other interventions beyond exercise or usual care
Outcomes	Pain intensity was assessed using the Visual Analogue Scale (VAS). Studies were required to report VAS scores at both baseline and post-intervention assessments	Studies without VAS-based pain assessments, without available pre- and post-intervention data, or without sufficient statistical information to calculate effect sizes
Study design	Only randomized controlled trials were included; for studies reporting the same trial, only the one with the largest sample size was included	Non- randomized controlled trials, such as quasi-experimental and observational studies

### 2.4 Outcome

Pain intensity was assessed using the Visual Analogue Scale (VAS), a widely used and validated instrument for evaluating subjective pain severity (Begum and Hossain, 2019). The VAS typically consisted of a 100-mm horizontal line anchored by two endpoints, ranging from 0 (“no pain”) to 10 (“worst imaginable pain”). Higher scores indicated greater pain intensity. For studies that assessed pain intensity using a 0–100 VAS, the reported scores were divided by 10 to convert them to an equivalent 0–10 scale before analysis, thereby ensuring comparability across studies. According to the IMMPACT recommendations, a reduction of 1 point on the 0–10 pain intensity scale was considered the minimal clinically important difference (MCID) (Dworkin et al., 2008).

### 2.5 Data extraction and coding

Two reviewers (SXJ and CJ) independently extracted data using a standardized Excel template. The extracted information comprised study characteristics (e.g., author, publication year, and geographical location), participant characteristics (e.g., age and sample size), intervention characteristics (e.g., exercise modality, frequency, duration, and intervention length), comparator characteristics (e.g., control type and sample size), and outcome data (e.g., reported findings). Mean values and standard deviations (SDs) for VAS pain intensity were extracted at baseline and post-intervention for both the exercise and control groups. Any discrepancies were resolved through discussion between the two reviewers and, when necessary, adjudicated by a third reviewer (CQN). Mean change scores were calculated as the difference between pre-intervention and post-intervention values to quantify changes in pain intensity. When SDs of change scores were not directly reported, they were estimated using a correlation coefficient of 0.5 between baseline and post-intervention measurements, reflecting a commonly accepted assumption of measurement reliability (Chandler et al., 2019). Missing SDs were derived from reported confidence intervals, p-values, or t-values. When the required data were unavailable, the corresponding authors were contacted to obtain the missing information. Because the dose–response analysis required standard errors (SEs), reported SDs were converted to SEs using the following formula:


$$SE=\frac{SD}{\sqrt{N}}$$

where N represents the sample size.

The data were cross-checked for accuracy by the reviewers, and a third author (CQN) verified the final dataset.

To perform the dose-response network meta-analysis, interventions were classified according to a three-level framework. At the first level, all interventions were broadly divided into two categories: ‘Exercise’ and ‘Control’. At the second level, exercise interventions were grouped according to the exercise modality reported in the original studies, including ‘Aerobic Exercise’ (AE), ‘Aquatic Exercise’ (AQE), ‘Resistance Training’ (RT), ‘Balance Training’ (BT), ‘Flexibility/Stretching Training’, ‘Mind–Body Exercise’ (e.g., Tai Chi; MBE), and ‘Combined Exercise Training’ (CET), defined as a combination of two or more of the above-mentioned exercise modalities. **Table 2** provides detailed definitions of each exercise modality based on the American College of Sports Medicine (ACSM) guidelines (Ozemek et al., 2025).

**Table 2:** Definitions of different exercise modalities.

Exercise type	Definition
AE	AE refers to continuous or rhythmic physical activities that primarily rely on oxidative metabolism to sustain energy production and involve large muscle groups, leading to improvements in cardiorespiratory fitness. Typical examples include running, cycling, and walking
RT	RT involves exercises designed to improve muscular strength, endurance, or power by working against external resistance, such as free weights, resistance bands, or body weight
CET	A multimodal exercise program that incorporates two or more distinct exercise modalities or components, such as aerobic, resistance, flexibility, balance, or mind–body exercise, within the same intervention program
MBE	Exercise modalities that integrate physical movement, controlled breathing, and focused mental attention, such as yoga and Tai Chi, with the aim of promoting physical and psychological well-being
AQE	Exercise performed in a water-based environment, typically involving aerobic, resistance, flexibility, or other physical activities adapted to the properties of water, such as buoyancy and hydrostatic pressure
BT	Exercise interventions primarily designed to improve postural stability and balance control through activities that challenge the ability to maintain the body’s center of mass within the base of support, including static and dynamic balance exercises, weight-shifting, single-leg standing, and balance tasks performed on unstable surfaces
Flexibility/Stretching Training	Exercise interventions primarily designed to improve joint range of motion and musculoskeletal flexibility through systematic stretching or flexibility exercises, including static stretching, dynamic stretching, active or passive stretching, and related range-of-motion exercises

AE, Aerobic Exercise; AQE, Aquatic Exercise; BT, Balance Training; CET, Combined Exercise Training; Flexibility/Stretching, Flexibility/Stretching Training; MBE, Mind-Body Exercise; RT, Resistance Training.

At the third level, interventions were further classified according to weekly exercise dose, calculated as the product of exercise duration, frequency, and intensity and expressed as metabolic equivalent of task minutes per week (MET-min/week). Warm-up and cool-down periods were excluded from the calculation of total exercise duration. For interventions involving progressive changes in exercise duration during the intervention period, the average weekly exercise duration was calculated and used. Exercise intensity was coded based on the 2024 Adult Physical Activity Guidelines, taking into account the exercise modality and intensity indicators reported in the original studies, such as the percentage of maximal heart rate and rating of perceived exertion (Herrmann et al., 2024). This guideline provides MET values covering a wide range of exercise types, which were sufficient for allocating intensity in our analysis.

For MBE, including Tai Chi, Qigong, and yoga, intensity was coded based on the reported exercise characteristics and corresponding MET values from the 2024 Physical Activity Compendium. For AE, when intensity was reported as a percentage of maximal heart rate (%HRmax), exercise intensity was classified according to established ACSM guidelines: light intensity was defined as <64% HRmax, moderate intensity as 64–76% HRmax, vigorous intensity as 77–93% HRmax, and near-maximal to maximal intensity as ≥94% HRmax. These intensity categories were then matched with corresponding MET values from the 2024 Physical Activity Compendium. RT intensity followed ACSM guidelines based on %1 repetition maximum (1RM; light <50%, moderate 50–69%, vigorous ≥70–85%); if %1RM was unavailable, intensity was inferred from rating of perceived exertion (≈12–13 = moderate; 14–17 = vigorous) or repetition ranges (12–20, 8–12, 6–8 reps). When studies reported only qualitative intensity levels, such as “moderate, “ we applied the ACSM absolute-intensity thresholds (light ≈ 2.0–2.9 MET, moderate ≈ 3.0–5.9 MET, vigorous ≥6.0 MET) and used the midpoint of each range (e.g., 4.5 MET for moderate, 8.0 MET for vigorous). For studies lacking specific intensity details, we assigned generic codes from the 2024 Physical Activity Compendium, selecting conservative estimates to minimize potential bias (e.g., general intensity for walking was 3.5 MET). For combined exercise training, the total weekly exercise dose was calculated as the sum of the doses of each individual exercise component (e.g., aerobic, resistance, and balance exercises) included in the intervention program. For progressive exercise programs in which exercise intensity changed over time, the average exercise intensity across the entire intervention period was used to estimate the overall exercise dose.

Based on the estimated MET-min/week, each intervention was assigned to the nearest predefined dose category. The interventions were grouped into predefined categories ranging from 0 (usual care) to 1400 MET-min/week (0, 100, 150, 200, 250, 300, 350, 400, 450, 500, 550, 600, 700, 800, 1000, 1200, and 1400 MET-min/week). This categorization ensured adequate network connectivity while minimizing potential discrepancies between the assigned and actual intervention doses. Furthermore, for studies reporting exercise attendance, the dose categorization was adjusted in cases of lower attendance to reflect the reduced actual exercise dose, since the amount of exercise delivered in interventions is often lower than the prescribed or intended dose. For example, intensity of bicycling at a moderate pace is 7 MET. If the study reports that the exercise was performed three times a week for 30 minutes per session, the total exercise intensity would be calculated as follows: 7 (intensity) × 30 (minutes) × 3 (sessions) = 630 MET-min/week. If the study reports an average exercise session attendance rate of 80%, the dose would be adjusted to 630 × 0.8 = 504 MET-min/week. This adjusted dose would then be rounded to the nearest predefined dose category, which in this case would be 500 MET-min/week. Detailed procedures and illustrative examples of exercise dose calculation are provided in **Supplementary Content 2**.

### 2.6 Statistical analysis

#### 2.6.1 Dose-response network meta-analysis

As all included studies used the same outcome measurement instrument, mean differences (MD) were calculated using changes from baseline to post-intervention values in both the intervention and control groups. The dose-response association between exercise dose and different exercise modalities was examined using the ‘MBNMAdose’ package in R (version 4.4.3). Following the default specifications of ‘MBNMAdose’, vague (non-informative) prior distributions were adopted to minimize the influence of prior assumptions and allow the observed data to primarily determine the model estimates.

Before conducting the dose-response analysis, we evaluated the fundamental assumptions of network meta-analysis, including network connectivity, consistency of evidence, and transitivity across comparisons (Higgins et al., 2012; Dias et al., 2013; Donegan et al., 2013). Second, multiple dose-response functions were assessed to characterize the association between exercise dose and treatment effects, including Emax, restricted cubic spline, log-linear, and quadratic models. Model fit was compared using several criteria, including the deviance information criterion (DIC), residual standard deviation, model complexity, and residual deviance. Consistent with previous dose-response network meta-analysis studies, the model yielding the lowest DIC was considered to provide the best fit and was selected for subsequent analyses (Zhang et al., 2025; Cheng et al., 2026; Zhang et al., 2026a; Zhang et al., 2026b). Considering the expected between-study heterogeneity, a random-effects model was applied to account for variability among the included studies. The estimated dose-response parameters were used to identify the exercise doses associated with significant improvements in pain intensity and to estimate the relative ranking of different exercise modalities and dose levels based on their probability of producing beneficial effects (Pedder et al., 2019). In the Bayesian dose-response analyses, statistical significance was determined when the 95% credible interval (CrI) of the estimated effect did not include zero. Finally, based on the resulting effective dose ranges, we formulated exercise prescriptions with reference to standardized adult physical activity intensity classification tables (Herrmann et al., 2024).

#### 2.6.2 Additional analyses

Network meta-regression analyses were performed to investigate whether study-level characteristics modified the estimated intervention effects. Prespecified potential effect modifiers included mean age (years), intervention length (weeks), and body mass index (BMI). Regression coefficients with 95% CrI were estimated to evaluate the associations between these covariates and pain intensity; a CrI spanning zero was considered indicative of no statistically meaningful association. All network meta-regression models were fitted using the R package ‘MBNMAdose.’

Sensitivity analyses were conducted by excluding studies considered to have a high risk of bias to assess the robustness of the primary results. Potential publication bias and small-study effects were evaluated using funnel plot inspection, together with Begg’s and Egger’s statistical tests (Begg and Mazumdar, 1994; Lin et al., 2018). A visually symmetrical funnel plot and a p-value >0.05 were considered indicative of no substantial evidence of publication bias.

### 2.7 Risk of bias and certainty of evidence

Risk of bias was evaluated using the Cochrane Risk of Bias 2 (RoB 2) tool, which assesses five domains: the randomization process, deviations from intended interventions, missing outcome data, measurement of the outcome, and selection of the reported result. Each study was categorized as having a low risk of bias, some concerns, or a high risk of bias (Sterne et al., 2019). Two authors (SXJ, CJ) independently performed the assessment, with disagreements resolved by a third author (CQN).

The certainty of evidence for the network estimates was assessed using the confidence in network meta-analysis (CINeMA) web application, which evaluates six domains: within-study bias, reporting bias, indirectness, imprecision, heterogeneity, and incoherence (Nikolakopoulou et al., 2020). Evidence confidence was graded as high, moderate, low, or very low.

## 3 Results

### 3.1 Study selection

The database search identified 4834 records across four electronic databases. After removing 751 duplicates, 4083 titles and abstracts were screened, of which 3985 irrelevant records were excluded. Subsequently, 98 full-text articles were assessed for eligibility, and 77 were excluded based on the predefined inclusion and exclusion criteria. Thus, 21 studies met the eligibility criteria and were included in the systematic review. An additional three studies were identified through reference screening of relevant reviews, resulting in a total of 24 studies included in the analysis. The study selection process is illustrated in **Figure 1**.

**Figure 1:**
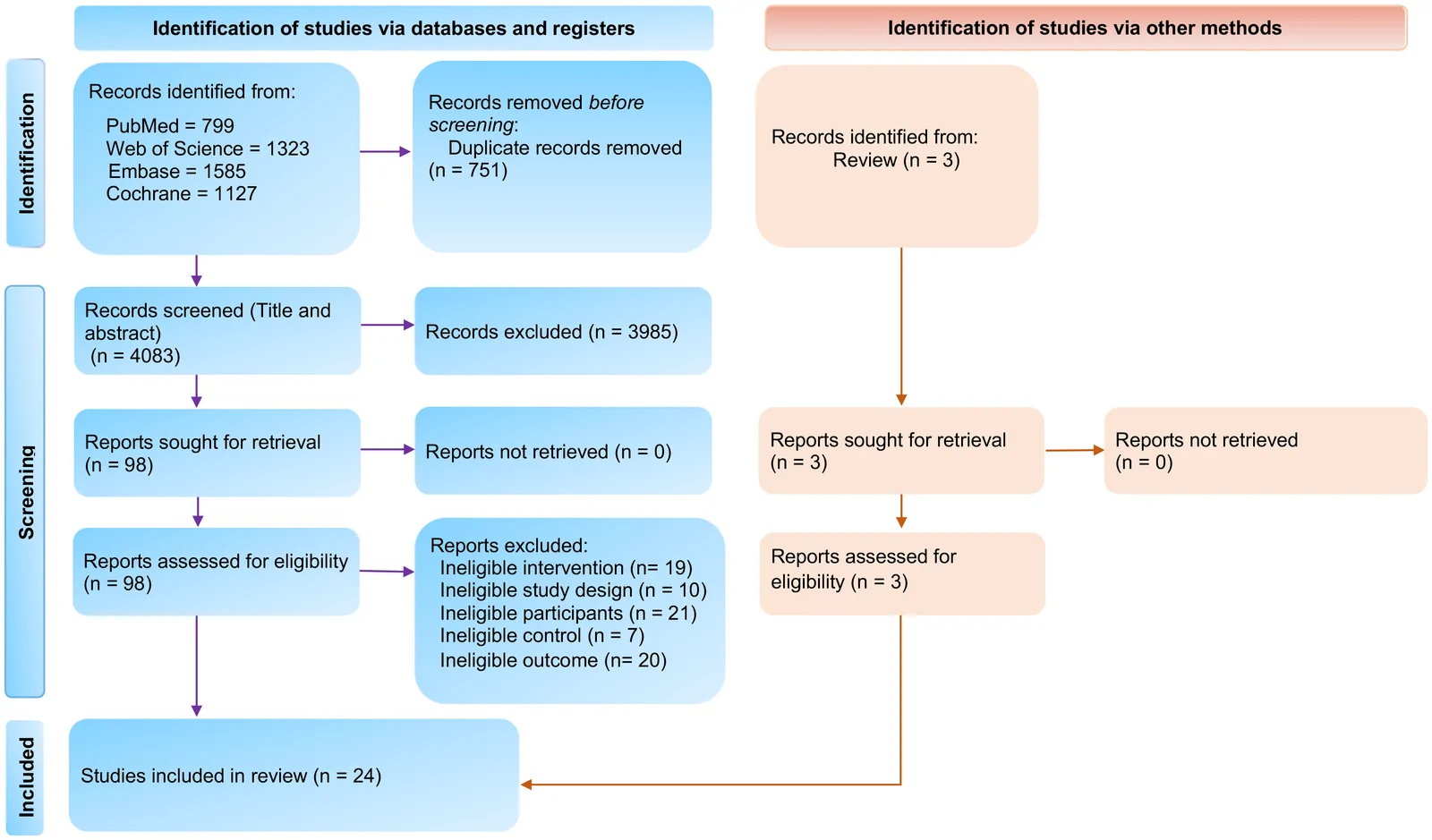
Screening and selection process of studies for the systematic review.

### 3.2 Study characteristics

A total of 1992 women with FMS participated in the included studies, with a mean age of 49.17 years. Regarding exercise modalities, MBE and AE were the most frequently represented interventions, each comprising eight arms, followed by flexibility/stretching (6 arms), RT (5 arms), CET (5 arms), AQE (3 arms), and BT (2 arms). Comparators included no-exercise controls, usual care, waiting-list controls, and other exercise interventions involving different modalities from the intervention group. The prescribed exercise doses ranged from 100 to 1400 MET-min/week, with intervention periods ranging from 4 to 24 weeks. Reported adherence rates ranged from 57.1% to 100%. Five adverse events were reported across the included studies, of which only two studies reported events considered potentially related to exercise, including fractures and pain (Schachter et al., 2003; Assumpção et al., 2018). Andrade et al. reported one withdrawal due to worsening pain during stretching training, with the causal relationship with the intervention rated as likely. Schachter et al. reported that one participant in the short-bout exercise group sustained a metatarsal stress fracture and subsequently withdrew from the study. The remaining withdrawals or adverse events were attributed to participants’ underlying conditions or unrelated personal reasons rather than the exercise interventions. The included studies were conducted across multiple regions, including Asia, Europe, and America. Detailed characteristics of the included studies are presented in **Supplementary Content 3**.

### 3.3 Risk of bias

Overall, three studies were rated as having a high risk of bias, primarily due to a high proportion of missing outcome data. Sixteen studies were judged as having some concerns, mainly arising from uncertainties in the randomization process, deviations from intended interventions, potential bias due to missing outcome data, insufficient information regarding outcome measurement, and potential concerns regarding the selection of the reported result. Five studies were classified as having a low risk of bias across all domains. Detailed risk-of-bias assessment results are presented in **Supplementary Content 4**.

### 3.4 Dose–response network meta-analysis

#### 3.4.1 Hypothesis testing and model selection

No evidence was found to suggest that the dose-response network meta-analysis violated the assumptions of connectivity (**Figure 2**; **Supplementary Content 5.1**), consistency (**Supplementary Content 5.2**), or transitivity (**Supplementary Content 5.3**). Ultimately, the quadratic function which had the lowest DIC, was selected as the best-fitting approach (**Supplementary Content 5.4**).

**Figure 2:**
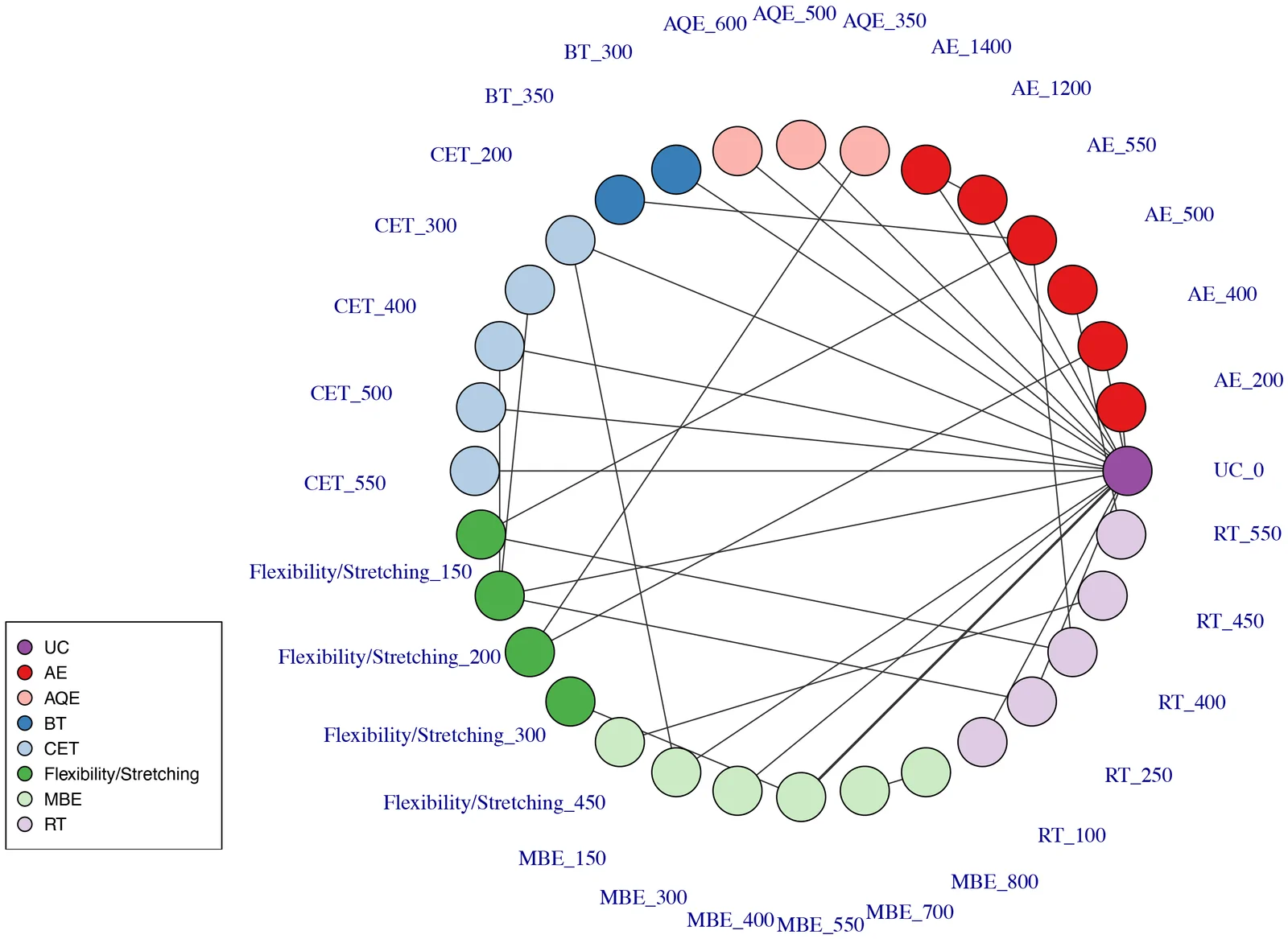
Dose-level network. Line thickness represents the number of studies available for comparison. The first value represents the specific intervention, and the second value corresponds to the dose (MET-min/week). AE, Aerobic Exercise; AQE, Aquatic Exercise; BT, Balance Training; CET, Combined Exercise Training; Flexibility/Stretching, Flexibility/Stretching Training; MBE, Mind-Body Exercise; RT, Resistance Training; UC, Usual Care.

#### 3.4.2 Dose-response relationship between exercise and pain intensity

**Table 3** systematically presents the results of the dose–response network meta-analysis. Our analysis identified a positive dose–response association between total exercise dose and pain improvement in women with FMS, with greater exercise doses associated with greater improvements in pain. A significant improvement was observed at 190 MET-min/week, with the MCID reached at 340 MET-min/week, while the greatest estimated effect within the evaluated dose range observed at 1400 MET-min/week (MD = −3.02, 95% CrI: −5.42, −0.52; **Figure 3**; **Supplementary Content 5.5**). Sensitivity analysis (**Supplementary Content 5.6**), excluding studies at high risk of bias, showed that the minimum effective dose increased to 240 MET-min/week, while the dose associated with the maximum effect remained unchanged at 1400 MET-min/week. The corresponding treatment effect was slightly greater (MD = −3.09, 95% CrI: −5.71, −0.44).

**Table 3:** Summary of dose-response network meta-analysis.

Category	Group	Arms^a^	Effective dose (MET-min/week)^b^	Maximum effects dose (MET-min/week)^c^	Mean difference and 95% CrI (maximum effects dose)
Overall results^d^	All exercise modalities	37	190-1400	1400	-3.02 (-5.42, -0.52)
Stratified by exercise modality	CET	5	NA	NA	NA
	AQE	3	NA	NA	NA
	AE	8	290-1400	1400	-2.93 (-5.36, -0.49)
	RT	5	400-550	550	-1.81 (-3.6, -0.06)
	MBE	8	140-800	800	-3.07 (-4.99, -1.09)
	Flexibility/stretching	6	NA	NA	NA
	Balance	2	NA	NA	NA
Sensitivity analyses	Excluding high risk bias studies	32	240-1400	1400	-3.09 (-5.71, -0.44)

a The number of exercise arms included in each analysis. ^b^Model-identified effective dose (MET-min/week), calculated as exercise intensity × duration × frequency. ^c^Model-identified dose associated with the maximum effect (MET-min/week), calculated as exercise intensity × duration × frequency. ^d^Dose–response results for overall exercise and pain. NA, Not Applicable (no statistically significant effect observed). AE, Aerobic Exercise; AQE, Aquatic Exercise; Balance, Balance/Proprioceptive Training; CET, Combined Exercise Training; flexibility/stretching, Flexibility/Stretching Training; MBE, Mind-Body Exercise; RT, Resistance Training.

**Figure 3:**
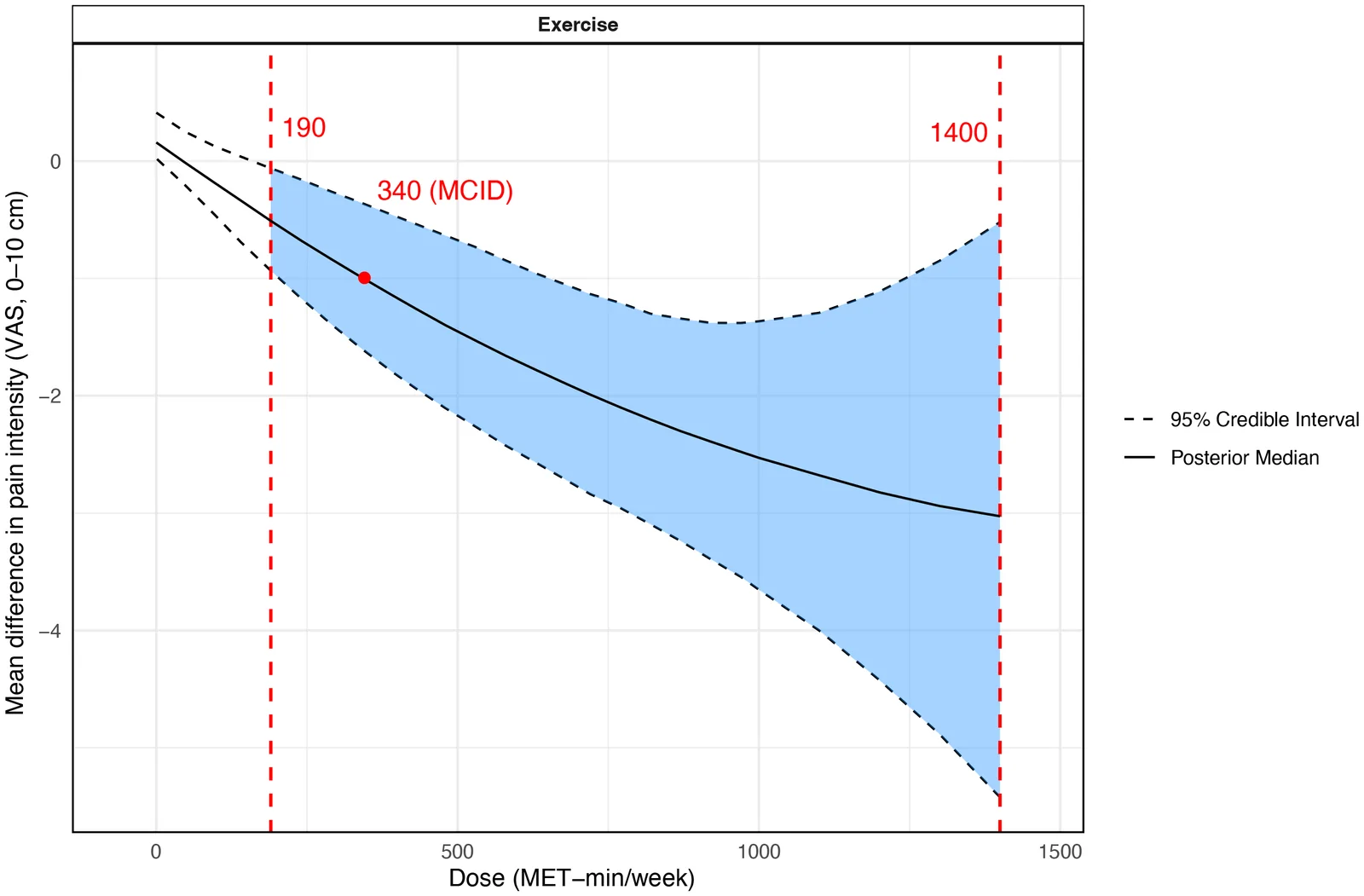
Dose–response association between total exercise dose (MET-min/week) and pain intensity in adult women with fibromyalgia syndrome. The red dashed lines represent the minimum and maximum effective doses (190–1400 MET-min/week), the blue shaded area indicates the significant region, and the red dots indicate the dose corresponding to the minimal clinically important difference (340 MET-min/week). MET-min/week, Metabolic Equivalent of Task minutes per week; MCID, Minimal Clinically Important Difference.

**Figure 4** illustrates the dose–response associations between different exercise modalities and pain intensity. Significant dose–response relationships were identified for AE, RT, and MBE, whereas significant associations were not detected for CET, AQE, BT, and Flexibility/Stretching Training, potentially due to limited available evidence. For AE, the effective dose range was 290–1400 MET-min/week, with the MCID reached at 310 MET-min/week, while the dose associated with the maximum effect was identified at 1400 MET-min/week (MD = −2.93, 95% CrI: −5.36, −0.49). For RT, the effective dose range was 400–550 MET-min/week, with the greatest improvement observed at 550 MET-min/week (MD = −1.81, 95% CrI: −3.60, −0.06); however, the dose corresponding to the MCID was not within the statistically significant effective dose range. For MBE, the effective dose range was 140–800 MET-min/week, with the MCID reached at 230 MET-min/week, while the dose associated with the maximum effect was identified at 800 MET-min/week (MD = −3.07, 95% CrI: −4.99, −1.09). The overall predicted ranking of exercise modalities and doses indicated that MBE at 800 MET-min/week was the most effective intervention–dose combination (**Supplementary Content 5.7**).

**Figure 4:**
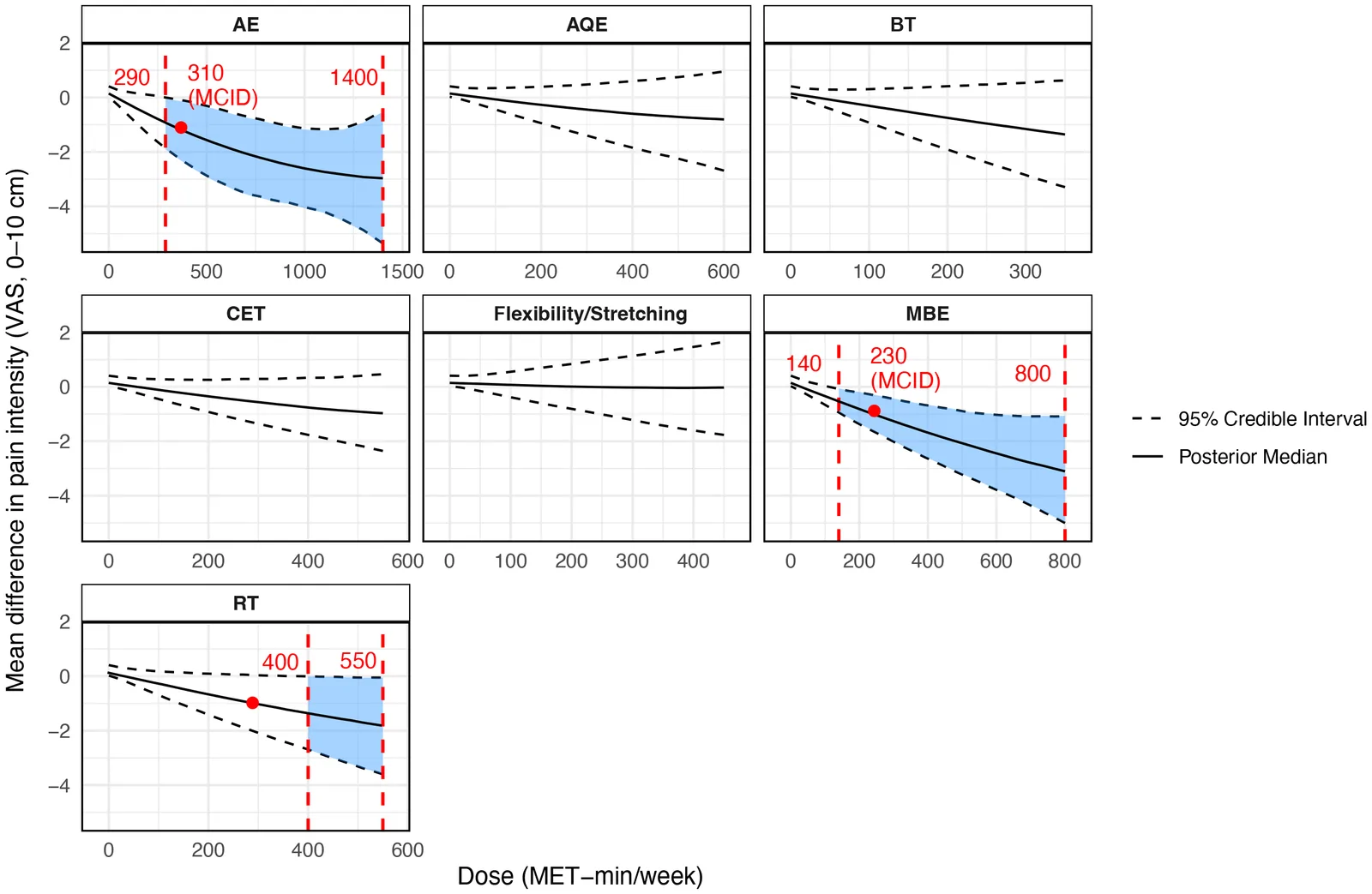
Dose–response association between different exercise dose (MET-min/week) and pain intensity in adult women with fibromyalgia syndrome. The red dashed lines represent the minimum and maximum effective doses, the blue shaded area indicates the significant region, and the red dots indicate the dose corresponding to the minimal clinically important difference. MET-min/week: Metabolic Equivalent of Task minutes per week; AE: Aerobic Exercise; AQE: Aquatic Exercise; BT: Balance Training; CET, Combined Exercise Training; Flexibility/Stretching, Flexibility/Stretching Training; MBE, Mind-Body Exercise; RT, Resistance Training; MCID, Minimal Clinically Important Difference.

Network meta-regression analyses showed that none of the evaluated covariates, including age, intervention length, and BMI, significantly modified the effects of exercise interventions on pain intensity (**Supplementary Content 6**).

### 3.5 Translation of exercise doses

**Table 4** presents the effective and model-identified maximum-effect weekly exercise doses for three exercise modalities for improving pain among women with FMS. For illustrative interpretation, the corresponding weekly exercise durations were estimated by converting the prescribed MET-min/week into minutes based on the estimated energy expenditure of each exercise modality and rounding to the nearest whole minute.

**Table 4:** Translation of model-derived exercise doses.

Exercise modality	Effective dose (MET-min/week)	Maximum effects dose (MET-min/week)	Representative activity	MET value and source	Effective accumulation (min/week)	Optimal accumulation (min/week)	Confidence in evidence
AE	290-1400	1400	Aerobic, general	7.3 (code 02000)	40-195	190	Very low
AE	290-1400	1400	Jogging, in place	4.8 (code 12025)	60-290	290	Very low
AE	290-1400	1400	Walking, for pleasure	3.5 (code 17250)	80-400	400	Very low
RT	400-550	550	Resistance training, multiple exercises	3.5 (code 02054)	115-155	160	Moderate
MBE	140-800	800	Tai chi/qigong, general	3.3 (code 15670)	40-240	240	Low

The representative activities were selected based on the representative exercises reported in the included primary studies. These minute-based conversions are provided for illustrative interpretation of the estimated exercise doses only and should not be considered as validated clinical prescriptions or absolute recommendations. Only exercise modalities with a model-identified effective dose or maximum effects dose in **Table 3** are included. Recommended accumulation was calculated as dose (MET-min/week) divided by the representative MET value and rounded to the nearest minute. The representative activities were selected based on the representative exercises reported in the included primary studies. 3–6 MET represents moderate-intensity activity, and >6 MET represents high-intensity activity, serving as a reference for absolute exercise intensity. Confidence ratings were retained from the available CINeMA/GRADE results. AE, Aerobic Exercise; MBE, Mind-Body Exercise; RT, Resistance Training; MET-min/week, Metabolic Equivalent of Task minutes per week.

### 3.6 Publication bias and certainty of evidence

Detailed assessments of publication bias are presented in **Supplementary Content 7**. The funnel plot showed no evident asymmetry, and neither Egger’s nor Begg’s tests detected significant small-study effects (p > 0.05), suggesting that the findings were unlikely to be substantially affected by publication bias.

The CINeMA assessment indicated that the certainty of evidence for all exercise interventions ranged from very low to moderate. The evidence was primarily downgraded due to within-study bias, heterogeneity, imprecision, and incoherence across network comparisons (**Supplementary Content 8**).

## 4 Discussion

### 4.1 Principal findings and implications

This study systematically evaluated the comparative effects of different exercise modalities and doses on pain among women with FMS. We found a postive dose–response association between overall exercise dose and pain improvement, with greater benefits observed at higher doses, reaching the greatest effect at approximately 1400 MET-min/week. AE, MBE, and RT were all associated with significant improvements in pain, although the effective dose ranges and doses associated with the maximum effects varied across modalities. Among the evaluated interventions, MBE showed the greatest improvement at approximately 800 MET-min/week, whereas AE and RT demonstrated their greatest effects at 1400 and 550 MET-min/week, respectively. These findings suggest that the dose associated with the maximum effect for pain management may differ according to exercise modality, highlighting the importance of considering both exercise type and dose when developing individualized exercise prescriptions for women with FMS. Meta-regression analyses revealed no significant effect modifiers, indicating that the overall results were unlikely to be meaningfully affected by study-level characteristics. The sensitivity analyses also produced comparable findings, further supporting the robustness of the primary results.

These findings have two important clinical implications. First, they may assist clinicians in tailoring exercise prescriptions to improve pain management in women with FMS. Second, the dose-specific estimates identified in this study may provide a useful basis for designing future intervention trials and validating the effectiveness of different exercise doses, thereby contributing to a stronger evidence base for exercise prescription in FMS.

### 4.2 Interpretation and comparison with existing literature

Our findings showed that MBE provided the greatest improvement in pain, consistent with the findings of a recent systematic review (Pan et al., 2026). Building on this evidence, our study further identified the effective dose range and the dose associated with the maximum effect of MBE for pain management in women with FMS. The beneficial effects of MBE may be attributable to its combined effects on central pain modulation, autonomic regulation, stress reduction, and psychological well-being, which may help reduce pain sensitivity and improve pain perception in individuals with FMS (Durusoy and Ünal, 2025). However, most of the MBE interventions included in our analysis were Tai Chi and Qigong. Future studies should therefore investigate other MBE modalities, such as yoga and Pilates, to broaden the evidence base and further clarify the effects of different MBE modalities on pain in women with FMS.

Regarding AE, two previous studies have demonstrated the beneficial effects of AE alone on pain relief in individuals with FMS (Casanova‐Rodríguez et al., 2025; Li et al., 2025), which is consistent with our findings. Several mechanisms may underlie the analgesic effects of AE in individuals with FMS. First, AE may activate exercise-induced hypoalgesia (EIH). Moderate-intensity, sustained AE can stimulate the release of endogenous opioids, including endorphins, thereby increasing pain thresholds, reducing pain sensitivity, and attenuating central sensitization. Importantly, previous research suggests that the EIH response remains responsive in individuals with FMS (Martins-Pinge, 2011). Second, AE may influence neurotransmitter activity and neural regulation, which may contribute to improved endogenous pain modulation and reduced pain perception (Heijnen et al., 2016). Collectively, these physiological responses may help explain the observed analgesic effects of AE in individuals with FMS.

Regarding RT, recent evidence has demonstrated its beneficial effects on pain reduction in individuals with FMS, with greater improvements observed with higher exercise intensity and frequency (Casanova-Rodríguez et al., 2026). However, the light-intensity group approached the null hypothesis and showed high heterogeneity, warranting cautious interpretation (Casanova-Rodríguez et al., 2026). Our dose–response analysis further showed that RT produced a significant analgesic effect only when the exercise dose reached ≥400 MET-min/week, suggesting a potential minimum dose required for meaningful pain reduction. The analgesic effects of RT may be attributed to enhanced exercise-induced hypoalgesia, improved descending pain inhibition, and reductions in muscular fatigue and peripheral nociceptive input (Busch et al., 2013). In addition, other exercise modalities, such as sensorimotor training, have also been suggested to potentially alleviate pain in individuals with FMS, although their dose–response relationships remain to be further investigated (Parisi et al., 2025).

Finally, although our analysis did not identify significant effects of CET, AQE, flexibility/stretching, or BT on pain, these findings should not be interpreted as evidence that these exercise modalities are ineffective in individuals with FMS. The available evidence for these modalities was limited, which may have reduced the statistical power and increased the uncertainty of the estimates. Further well-designed RCTs with larger samples, standardized intervention protocols, and adequately reported exercise doses are warranted to clarify their effects and dose–response relationships. Moreover, the dose–response relationships may change as additional studies become available. Thus, the current findings should be regarded as provisional and subject to refinement as the evidence base expands.

### 4.3 Clinical recommendations

From a clinical perspective, the clinical significance of the observed effects should also be considered. The Initiative on Methods, Measurement, and Pain Assessment in Clinical Trials (IMMPACT) recommends that, for 0–10 numerical pain rating scales in chronic pain trials, reductions of 10%–20% represent minimal important improvement, >30% represent moderate improvement, and >50% represent substantial improvement (Dworkin et al., 2008). Based on these benchmarks, RT at the dose associated with the greatest estimated effect within the evaluated dose range achieved a minimally important reduction in pain, although with substantial uncertainty. AE and MBE achieved moderately important reductions in pain at their corresponding doses associated with the greatest estimated effects. Notably, these levels of improvement were observed only at specific dose points with the greatest estimated benefits, whereas the broader effective dose ranges remained subject to considerable uncertainty, with credible intervals approaching the null effect. Although greater pain relief may be achieved with higher exercise doses, increasing exercise volume may be constrained by adherence, fatigue, time commitment, and overall feasibility, particularly in individuals with FMS. Notably, MBE imposes relatively low physical demands and was associated with its maximum effect at approximately 240 min/week, suggesting a potentially favorable balance between effectiveness and feasibility. Therefore, exercise prescriptions should consider effectiveness, safety, and feasibility rather than simply targeting the dose associated with the maximum effect. Regarding intervention length, although we did not identify a significant moderating effect of intervention length, the included studies had a mean intervention length of approximately 13 weeks. Maintaining an intervention period of around 12–13 weeks may therefore be a reasonable practical consideration when designing exercise programs for pain management in individuals with FMS, while recognizing that the optimal length remains uncertain.

### 4.4 Strength and limitations

To the best of our knowledge, this study is the first to systematically investigate the dose–response relationships between different exercise modalities and pain in women with FMS. A major strength of this study was the use of MET-min/week to quantify exercise dose, integrating exercise intensity, duration, and frequency into a common metric and enabling their incorporation into a unified network framework for comparative analysis. In contrast, conventional subgroup analyses often focus on a single dimension of exercise exposure, such as intervention frequency, while failing to account for other key components of exercise dose. We also conducted meta-regression analyses to examine whether potential study-level covariates modified the observed effects. By combining direct and indirect evidence within a Bayesian framework and modeling dose–response relationships, this study moves beyond simply determining whether exercise is beneficial and provides insights into which exercise modalities and doses may offer greater benefits for pain management in women with FMS.

Despite these strengths, several limitations should be acknowledged. First, although MET-min/week provides a standardized approach to quantifying exercise dose by integrating intensity, duration, and frequency, this metric also has inherent limitations. MET estimates are based on standardized assumptions about resting metabolic expenditure and may not fully capture individual differences in age, sex, body composition, functional status, and health conditions. Moreover, standardized MET values may not accurately reflect exercises with fluctuating intensities or individual physiological responses to exercise. As MET-based doses were estimated rather than directly measured, the calculated exercise doses may contain some degree of measurement error, which should be considered when interpreting the dose–response findings. Second, we applied strict eligibility criteria and excluded studies with insufficient or unclear information on exercise dose. Although this approach enhanced the reliability and interpretability of the dose–response analyses, it may have reduced the number of eligible studies and limited the breadth and generalizability of the available evidence. Third, the relatively small number of studies available for some exercise modalities and dose categories may have reduced the precision of the estimated dose–response relationships. In addition, for some exercise types, the dose associated with the greatest estimated effect was identified at the upper boundary of the evaluated dose range, suggesting that the current findings may be influenced by the limited distribution of available exercise doses. Future studies, particularly those evaluating higher exercise doses, are needed to further clarify the shape of the dose–response relationship and determine whether the observed pattern persists beyond the current evidence range. Finally, the certainty of evidence for several exercise modalities was rated as very low. Although this does not directly invalidate the dose–response estimates, the findings should be interpreted cautiously given the limited evidence base and potential risk of bias in some included studies. In addition, clinical heterogeneity, including differences in exercise modalities, session structures, supervision, progression strategies, intensity prescription methods, adherence, baseline pain severity, and participant characteristics, may have contributed to variability in the observed effects and dose–response relationships. Although MET-min/week provides a pragmatic approach for harmonizing exercise doses across studies, it may not fully capture the multidimensional characteristics of exercise interventions. These factors should be considered when interpreting the current findings and applying them to clinical practice. Future high-quality RCTs are needed to further validate these findings and refine the current evidence on exercise dose–response relationships for pain management in women with FMS.

## 5 Conclusions

This study systematically evaluated the comparative effects of different exercise modalities and doses on pain intensity in women with FMS. We identified a postive dose–response association between overall exercise dose and pain improvement, with greater benefits observed at higher doses. When stratified by exercise modality, AE, MBE, and RT were associated with significant improvements in pain, although the effective dose ranges and doses associated with the maximum effects varied across modalities. Among these interventions, MBE demonstrated the greatest improvement at approximately 800 MET-min/week, while AE and RT showed their greatest effects at 1400 and 550 MET-min/week, respectively. Given the limited number of available studies, it remains unclear whether the lack of significant effects for AQE and BT reflects true absence of benefit or insufficient statistical power due to limited evidence.

These findings provide evidence to inform the development of individualized, dose-specific exercise prescriptions for pain management in women with FMS. However, considering the overall risk of bias and the very low-to-moderate certainty of evidence, these identified doses should be regarded as exploratory estimates requiring further validation rather than definitive exercise prescriptions.

## Data Availability

The original contributions presented in the study are included in the article/**Supplementary Material**. Further inquiries can be directed to the corresponding author.
