# Supplementary material for: Comparative effectiveness of exercise types and doses for improving pain intensity in women with fibromyalgia syndrome: a systematic review and dose–response meta-analysis
Source: Front Physiol. 2026 Oct 28;17:1971006. doi: 10.3389/fphys.2026.1971006 (PMC13751228; doi:10.3389/fphys.2026.1971006)
Supplement: Supplementary file 1 [file DataSheet1.pdf]

Supplementary content

1. SEARCH STRATEGY 2
1.1 PUBMED 2
1.2 WEB OF SCIENCE 2
1.3 EMBASE 3
1.4 COCHRANE 3
2. EXERCISE INTERVENTION CLASSIFICATION AND WEEKLY DOSE CODING 5
3. STUDY CHARACTERISTICS 6
4. RISK OF BIAS 11
5. DOSE-RESPONSE NETWORK META-ANALYSIS 13
5.1 NETWORK CONNECTIVITY 13
5.2 CONSISTENCY 13
5.3 TRANSITIVITY 14
5.4 DOSE-RESPONSE MODEL SELECTION 19
5.5 DOSE-RESPONSE RELATIONSHIPS 24
5.6 SENSITIVITY ANALYSIS 25
5.7 RELATIVE EFFECTIVENESS RANKING 26
6. META-REGRESSION ANALYSIS 31
7. PUBLICATION BIAS 32
8. CONFIDENCE IN THE EVIDENCE USING CINEMA 33
8.1 WITHIN-STUDY BIAS 33
8.2 REPORTING BIAS 33
8.3 INDIRECTNESS 33
8.4 IMPRECISION 33
8.5 HETEROGENEITY 34
8.6 INCOHERENCE 34
8.7 OVERALL CINEMA JUDGMENT 34
REFERENCES 37

1. Search strategy

1.1 Pubmed

("Fibromyalgia"[Mesh] OR fibromyalgia[Title/Abstract] OR "fibromyalgia syndrome"[Title/Abstract] OR fibrositis[Title/Abstract]) AND ("Exercise"[Mesh] OR "Exercise Therapy"[Mesh] OR "Resistance Training"[Mesh] OR "Physical Fitness"[Mesh] OR "Motor Activity"[Mesh] OR "Walking"[Mesh] OR "Swimming"[Mesh] OR "Yoga"[Mesh] OR "Tai Ji"[Mesh] OR exercis*[Title/Abstract] OR "exercise therapy"[Title/Abstract] OR "physical activity"[Title/Abstract] OR "physical exercise"[Title/Abstract] OR "aerobic exercise"[Title/Abstract] OR "aerobic training"[Title/Abstract] OR "resistance exercise"[Title/Abstract] OR "resistance training"[Title/Abstract] OR "strength training"[Title/Abstract] OR stretching[Title/Abstract] OR flexibility[Title/Abstract] OR "balance training"[Title/Abstract] OR "aquatic exercise"[Title/Abstract] OR "water-based exercise"[Title/Abstract] OR hydrotherapy[Title/Abstract] OR swimming[Title/Abstract] OR walking[Title/Abstract] OR cycling[Title/Abstract] OR yoga[Title/Abstract] OR "tai chi"[Title/Abstract] OR "tai ji"[Title/Abstract] OR qigong[Title/Abstract] OR pilates[Title/Abstract] OR dance[Title/Abstract] OR dancing[Title/Abstract] OR "whole body vibration"[Title/Abstract] OR "whole-body vibration"[Title/Abstract] OR "vibration training"[Title/Abstract] OR multicomponent[Title/Abstract] OR "multi-component"[Title/Abstract] OR "combined exercise"[Title/Abstract] OR "combined training"[Title/Abstract] OR HIIT[Title/Abstract] OR "high-intensity interval training"[Title/Abstract] OR MICT[Title/Abstract] OR "moderate-intensity continuous training"[Title/Abstract]) AND (Randomized Controlled Trial[Publication Type] OR Controlled Clinical Trial[Publication Type] OR randomized[Title/Abstract] OR randomised[Title/Abstract] OR randomly[Title/Abstract] OR RCT[Title/Abstract] OR RCTs[Title/Abstract] OR "randomized trial"[Title/Abstract] OR "randomised trial"[Title/Abstract] OR "controlled clinical trial"[Title/Abstract] OR "clinical trial"[Title/Abstract] OR "intervention trial"[Title/Abstract] OR trial[Title/Abstract])

1.2 Web of Science

TS=(fibromyalgia OR "fibromyalgia syndrome" OR fibrositis) AND TS=(exercis* OR "exercise therapy" OR "physical activity" OR "physical exercise" OR "aerobic exercise" OR "aerobic training" OR "resistance exercise" OR "resistance training" OR "strength training" OR stretching OR flexibility OR "balance training" OR "aquatic exercise" OR "water-based exercise" OR hydrotherapy OR swimming OR walking OR cycling OR yoga OR "tai chi" OR "tai ji" OR qigong OR pilates OR dance OR dancing OR "whole body vibration" OR "whole-body vibration" OR "vibration training" OR multicomponent OR "multi-component" OR "combined exercise" OR "combined training" OR HIIT OR "high-intensity interval training" OR MICT OR "moderate-intensity continuous training") AND TS=(randomized OR randomised OR randomly OR RCT OR RCTs OR "randomized trial" OR "randomised trial" OR "controlled clinical trial" OR "clinical trial" OR "intervention trial" OR trial OR placebo)

1.3 Embase

('fibromyalgia'/exp OR fibromyalgia:ti,ab OR 'fibromyalgia syndrome':ti,ab OR fibrositis:ti,ab) AND ('exercise'/exp OR 'exercise therapy'/exp OR 'physical activity'/exp OR 'resistance training'/exp OR 'aerobic exercise'/exp OR 'walking'/exp OR 'swimming'/exp OR 'hydrotherapy'/exp OR 'yoga'/exp OR 'tai chi'/exp OR exercis*:ti,ab OR 'exercise therapy':ti,ab OR 'physical activity':ti,ab OR 'physical exercise':ti,ab OR 'aerobic exercise':ti,ab OR 'aerobic training':ti,ab OR 'resistance exercise':ti,ab OR 'resistance training':ti,ab OR 'strength training':ti,ab OR stretching:ti,ab OR flexibility:ti,ab OR 'balance training':ti,ab OR 'aquatic exercise':ti,ab OR 'water-based exercise':ti,ab OR hydrotherapy:ti,ab OR swimming:ti,ab OR walking:ti,ab OR cycling:ti,ab OR yoga:ti,ab OR 'tai chi':ti,ab OR 'tai ji':ti,ab OR qigong:ti,ab OR pilates:ti,ab OR dance:ti,ab OR dancing:ti,ab OR 'whole body vibration':ti,ab OR 'whole-body vibration':ti,ab OR 'vibration training':ti,ab OR multicomponent:ti,ab OR 'multi-component':ti,ab OR 'combined exercise':ti,ab OR 'combined training':ti,ab OR HIIT:ti,ab OR 'high-intensity interval training':ti,ab OR MICT:ti,ab OR 'moderate-intensity continuous training':ti,ab) AND ('randomized controlled trial'/exp OR 'controlled clinical trial'/exp OR randomized:ti,ab OR randomised:ti,ab OR randomly:ti,ab OR RCT:ti,ab OR RCTs:ti,ab OR 'randomized trial':ti,ab OR 'randomised trial':ti,ab OR 'controlled clinical trial':ti,ab OR 'clinical trial':ti,ab OR 'intervention trial':ti,ab OR trial:ti,ab OR placebo:ti,ab)

1.4 Cochrane

#1 MeSH descriptor: [Fibromyalgia] explode all trees
#2 fibromyalgia:ti,ab,kw OR (fibromyalgia syndrome):ti,ab,kw OR fibrositis:ti,ab,kw
#3 #1 OR #2
#4 MeSH descriptor: [Exercise] explode all trees
#5 MeSH descriptor: [Exercise Therapy] explode all trees
#6 MeSH descriptor: [Resistance Training] explode all trees
#7 MeSH descriptor: [Physical Fitness] explode all trees
#8 MeSH descriptor: [Motor Activity] explode all trees
#9 MeSH descriptor: [Walking] explode all trees
#10 MeSH descriptor: [Swimming] explode all trees
#11 MeSH descriptor: [Hydrotherapy] explode all trees
#12 MeSH descriptor: [Yoga] explode all trees
#13 MeSH descriptor: [Tai Ji] explode all trees
#14 exercise*:ti,ab,kw OR (exercise therapy):ti,ab,kw OR (physical activity):ti,ab,kw OR (physical exercise):ti,ab,kw OR (therapeutic exercise):ti,ab,kw OR (aerobic exercise):ti,ab,kw OR (aerobic training):ti,ab,kw OR (endurance training):ti,ab,kw OR (resistance exercise):ti,ab,kw OR (resistance training):ti,ab,kw OR (strength exercise):ti,ab,kw OR (strength training):ti,ab,kw OR stretching:ti,ab,kw OR flexibility:ti,ab,kw OR (flexibility exercise):ti,ab,kw OR (balance training):ti,ab,kw OR (aquatic exercise):ti,ab,kw OR (aquatic training):ti,ab,kw OR (water-based exercise):ti,ab,kw OR (pool-based exercise):ti,ab,kw OR hydrotherapy:ti,ab,kw OR balneotherapy:ti,ab,kw OR swimming:ti,ab,kw OR walking:ti,ab,kw OR cycling:ti,ab,kw OR yoga:ti,ab,kw OR (tai chi):ti,ab,kw OR (tai ji):ti,ab,kw OR qigong:ti,ab,kw OR pilates:ti,ab,kw OR dance:ti,ab,kw OR dancing:ti,ab,kw OR (mind-body exercise):ti,ab,kw OR (whole body vibration):ti,ab,kw OR (whole-body

vibration):ti,ab,kw OR (vibration training):ti,ab,kw OR multicomponent:ti,ab,kw OR (multi-component exercise):ti,ab,kw OR (combined exercise):ti,ab,kw OR (combined training):ti,ab,kw OR HIIT:ti,ab,kw OR (high-intensity interval training):ti,ab,kw OR MICT:ti,ab,kw OR (moderate-intensity continuous training):ti,ab,kw
#15 #4 OR #5 OR #6 OR #7 OR #8 OR #9 OR #10 OR #11 OR #12 OR #13 OR #14
#16 (randomized OR randomised OR randomly OR randomization OR randomisation OR RCT OR RCTs OR trial OR placebo OR (controlled clinical trial) OR (randomized controlled trial) OR (randomised controlled trial) OR (clinical trial) OR (intervention trial)):ti,ab,kw
#17 #3 AND #15 AND #16

2. Exercise intervention classification and weekly dose coding

- **Agent:** The agent level.
- **METs_min:** The energy expenditure per min in the study.
- **Min_session：** The duration of a single exercise intervention.
- **Frequency:** The number of times participants engaged in physical activity (times/week).
- **Exact_dose:** The exact estimated METs accumulated per week by participants (METs-min/week).
- **Dose:** The dose group by approximation (METs-min/week).
- **AE:** Aerobic exercise
- **CG:** Control group

Table S1. Dose categorization table

Study ID	Agent	METs_min	Min_session	Frequency	Prescribed dose	Average adherence rate (%)	Exact_dose	Dose
1	AE	7	30	3	630	80	504	500
1	CG	0	0	0	0	0	0	0

3. Study characteristics

Table S2. Study characteristics of the included studies.

Study (Author, year)	Intervention (n)	Length (weeks)	Age, years mean (SD)	BMI mean (SD)	Adherence (%)	Country	Frequency (week)	Duration (min)	Participants /Health Status	Detailed Exercise Protocol	Adverse Events
Altan et al. 2004 [1]	AQE (24)	12	43.14(6.39)	NR	96%	Turkey	3	35	Women with FMS (ACR 1990)	Aquatic mobility, stretching, walking/jumping, and relaxation; 37 C therapy pool.	1 withdrawal without stated reason
	CG (22)	12	43.91(6.26)	NR	88%	Turkey	0	0	Women with FMS (ACR 1990)	Warm-water immersion (37 C); no exercise.	2 hypertension, 2 arrhythmia (causality NR)
Altan et al. 2009 [2]	MBE (25)	12	48.20(6.5)	NR	100%	Turkey	3	60	Women with FMS (ACR 1990)	Supervised Pilates emphasizing posture, stretching, proprioception, and breathing; resistance bands and ball.	NR
	flexibility/stretching (24)	12	50.0(8.4)	NR	96%	Turkey	3	60	Women with FMS (ACR 1990)	Home relaxation plus dynamic and active/passive stretching; monthly telephone follow-up.	NR
Andrade et al. 2019 [3]	AQE (24)	16	48(8)	NR	89%	Brazil	2	45	Women with FMS (ACR 1990); low physical activity	Aquatic aerobic training; physiotherapist-supervised; HR/RPE individualized at VAT.	NR
	CG (24)	16	47(8)	NR	89%	Brazil	0	0	Women with FMS (ACR 1990); low physical activity	Usual daily activity; telephone monitoring every 15 days.	NR
Assumpcao et al. 2018 [4]	flexibility/stretching (14)	12	47.9(5.3)	28.9(4.2)	NR	Brazil	2	40	Women with FMS (ACR 1990), aged 30-55	Supervised whole-body stretching; 30-s holds, progressing from 3 to 5 repetitions.	1 withdrawal due to worsening pain; causal relationship with the intervention rated likely
	RT (16)	12	45.7(7.7)	28.1(4.7)	NR	Brazil	2	40	Women with FMS (ACR 1990), aged 30-55	Supervised progressive resistance training with dumbbells/weighted mats; 8 exercises x 8 repetitions; load increased at Borg 13.	NR
	CG (14)	12	46.9(6.5)	29.4(4.8)	NR	Brazil	0	0	Women with FMS (ACR 1990), aged 30-55	Usual pharmacological treatment; no exercise.	NR
Atan et al. 2020 [5]	AE (18)	6	46.57(9.41)	31.13(5.53)	94.7%	Turkey	5	50	Women with FMS (ACR 2016), aged ≥18	Cycle HIIT (4 x 4 min at 80-95% peak HR) plus brief resistance training and stretching.	NR

	AE (18)	6	47.36(8.01)	30.98(4.55)	94.7%	Turkey	5	60	Women with FMS (ACR 2016), aged ≥18	Continuous cycling (45 min at 65-70% peak HR) plus brief resistance training and stretching.	NR
	CG (14)	6	52.70(8.96)	32.69(3.03)	82.4%	Turkey	0	0	Women with FMS (ACR 2016), aged ≥18	Usual care; no supervised exercise.	NR
Baptista et al. 2012 [6]	MBE (38)	16	49.5	NR	95%	Brazil	2	60	Women with FMS (ACR 1990), aged 18-65	Supervised belly dance with warm-up and choreography; additional home practice from week 4.	NR
	CG (37)	16	49.1	NR	92.5%	Brazil	0	0	Women with FMS (ACR 1990), aged 18-65	Waitlist control; usual daily activity.	NR
Bircan et al. 2008 [7]	AE (13)	8	48.3(5.3)	NR	86.7%	Turkey	3	30	Women with FMS (ACR 1990)	Treadmill walking at 60-70% age-predicted maximal HR; light stretching before and after training.	NR
	RT (13)	8	46.0(8.5)	NR	86.7%	Turkey	3	40	Women with FMS (ACR 1990)	Progressive free-weight/body-weight resistance training; 4-12 repetitions; warm-up and cool-down stretching.	NR
Ekici et al. 2017 [8]	MBE (15)	4	37.13(6.37)	23.23(2.25)	71.4%	Turkey	3	60	Women with FMS (ACR 1990), aged >25; neck/shoulder pain	Supervised group Pilates; repetitions progressed from 5 to 10 with advanced movements.	1 withdrawal due to a health problem
	CG (21)	4	36.86(7.73)	22.48(3.12)	95.5%	Turkey	0	0	Women with FMS (ACR 1990), aged >25; neck/shoulder pain	Connective tissue massage; passive manual therapy without exercise.	NR
Fernandes et al. 2024 [9]	CET (41)	14	47.8(11.3)	29.1(4.8)	97.6%	Brazil	2	45	Women with FMS (ACR 1990), aged 18-65	Functional whole-body training with resistance bands; 2 sets x 10 repetitions; progression by perceived exertion.	NR
	flexibility/stretching (41)	14	55.0(8.0)	28.5(5.9)	92.7%	Brazil	2	45	Women with FMS (ACR 1990), aged 18-65	Whole-body stretching; 30-s holds x 3 sets; designed to avoid increasing HR.	NR
Garrido-Ardila et al. 2021 [10]	Balance (36)	5	56.06(8.37)	NR	NR	Spain	NR	NR	Women with clinician-diagnosed FMS, aged 18-71	Core-stability training emphasizing muscle activation, breathing, alignment, and movement control; physiotherapist-guided.	NR
	CG (33)	5	54.39(8.20)	NR	NR	Spain	0	0	Women with clinician-diagnosed FMS, aged 18-71	Usual medical care; no additional intervention.	NR

Hernando-Garijo et al. 2021 [11]	AE (14)	15	51.81(9.05)	27.25(7.30)	89.9%	Spain	2	50	Women with FMS (ACR 2016), aged 30-75	Video-guided low-impact aerobic telerehabilitation with mobility/stretching; intensity individualized to Borg 4-7.	NR
	CG (14)	15	55.06(8.51)	25.93(5.27)	82.4%	Spain	0	0	Women with FMS (ACR 2016), aged 30-75	Usual medical prescriptions; weekly video monitoring.	NR
Izquierdo-Alventosa et al. 2021 [12]	CET (16)	8	53.06(8.40)	NR	100%	Spain	2	60	Women with FMS (ACR 2016), aged 30-70	Low-intensity combined walking, resistance, and coordination training; Borg 1-4; progressive dumbbell loads.	NR
	CG (16)	8	55.13(7.35)	NR	100%	Spain	0	0	Women with FMS (ACR 2016), aged 30-70	Usual daily activity; no exercise intervention.	NR
Jones et al. 2008 [13]	CET (37)	24	49.62(7.65)	30.08(6.30)	93.3%	USA	3	60	Adults with FMS (ACR 1990), aged 18-65	Supervised aerobic, resistance, stretching, balance, and relaxation training; 40-50% maximal HR or Borg 10-12.	NR
	CG (38)	24	49.78(7.87)	30.22(6.54)	93.3%	USA	0	0	Adults with FMS (ACR 1990), aged 18-65	No exercise; telephone/home-visit monitoring; PYD or placebo continued.	NR
Kolak et al. 2022 [14]	AE (13)	12	48.3(10)	28.3(5)	NR	Turkiye	3	40	Women with FMS (ACR 2016), aged 18-65	Supervised treadmill walking plus stretching; 50-70% VO2max; CPET-based prescription.	NR
	RT (13)	12	46.0(11.2)	27.8(5.8)	NR	Turkiye	3	NR	Women with FMS (ACR 2016), aged 18-65	Supervised machine resistance training plus stretching; progressed from 50% to 70-80% 1RM.	NR
	flexibility/stretching (15)	12	45.9(7.6)	28.0(5)	NR	Turkiye	3	NR	Women with FMS (ACR 2016), aged 18-65	Home whole-body stretching; 30-s holds, 3-4 repetitions; telephone follow-up every 2 weeks.	NR
Ribeiro et al. 2021 [15]	WBVT (17)	6	56(6)	29.88(4.72)	100%	Brazil	3	3-10.6	Women with clinician-diagnosed FMS, aged 50-60; perimenopausal	Whole-body vibration with dynamic semi-squats; 35-40 Hz, 4 mm, 2.78-3.26 g; progressive sets/repetitions.	NR
	CG (15)	6	54(8)	29.79(3.44)	100%	Brazil	0	0	Women with clinician-diagnosed FMS, aged 50-60; perimenopausal	Usual daily activity; weekly telephone monitoring; no exercise.	NR
Rodriguez et al. 2012 [16]	AQE (19)	12	55.50(7.7)	NR	54.3%	Spain	2	60	Women with FMS (ACR), aged 18-65	Aquatic Biodanza with walking, slow limb movements, and stretching; approximately 29 C water.	NR

	flexibility/stretching (20)	12	55.30(7.5)	NR	57.1%	Spain	2	60	Women with FMS (ACR), aged 18-65	Land-based whole-body stretching.	NR
Rodriguez-Mansilla et al. 2021 [17]	CET (33)	4	52.24(6.19)	NR	70.2%	Spain	2	45	Women with clinician-diagnosed FMS, aged 30-65	Physiotherapist-guided mobility, walking, static balance, stretching, and controlled breathing.	NR
	MBE (31)	4	52.24(6.19)	NR	66%	Spain	2	45	Women with clinician-diagnosed FMS, aged 30-65	Qigong combining concentration, diaphragmatic breathing, balance, flexibility, and coordination.	NR
	CG (29)	4	52.24(6.19)	NR	61.7%	Spain	0	0	Women with clinician-diagnosed FMS, aged 30-65	Usual medical care; no exercise.	NR
Sarmento et al. 2020 [18]	MBE (10)	10	42.6(10.7)	NR	71.4%	USA	14	25	Women with FMS (ACR 2010/1990), aged 18-70; BMI ≤30	Six Healing Sounds Qigong: diaphragmatic breathing, gentle movements, meditation, and vocalization; home plus weekly group practice.	NR
	MBE (10)	10	56.1(12.3)	NR	71.4%	USA	14	25	Women with FMS (ACR 2010/1990), aged 18-70; BMI ≤30	Sham Qigong: gentle movements without healing sounds, synchronized breathing, or meditation; home plus weekly group practice.	NR
Sauch Valmaña et al. 2020 [19]	CET (23)	12	55.01(7.73)	NR	92%	Spain	2	90	Women with FMS (ICD-10-CM M79.7), aged 40-75	Aerobic, strength, and balance training; physiotherapist-guided; low intensity individualized to tolerance.	NR
	CG (25)	12	52.96(7.70)	NR	100%	Spain	0	0	Women with FMS (ICD-10-CM M79.7), aged 40-75	No exercise; questionnaire assessments only.	NR
Schachter et al. 2003 [20]	AE (51)	16	41.3(8.67)	NR	71%	Canada	4	30	Women with FMS (ACR 1990), aged 20-55; sedentary	Home video-guided low-impact aerobic dance; training segment progressed from 10 to 30 min; 126-144 bpm.	1 participant in the short-bout exercise (SBE) group sustained a metatarsal stress fracture and withdrew
	CG (36)	16	42.5(6.69)	NR	86%	Canada	0	0	Women with FMS (ACR 1990), aged 20-55; sedentary	Sedentary control; monthly group meetings; no planned exercise.	NR
Silva et al. 2019 [21]	RT (30)	12	44.93(10.30)	26.38(8.62)	88%	Brazil	2	40	Women with FMS (ACR 1990), aged 18-60	Supervised machine resistance training; 3 sets x 12 repetitions; progressed from 60% to 80% 1RM.	NR

	MBE (30)	12	49.40(8.30)	26.61(4.86)	88%	Brazil	2	40	Women with FMS (ACR 1990), aged 18-60	Physiotherapist-guided sophrology with relaxation, breathing awareness, and guided imagery.	NR
Valim et al. 2003 [22]	AE (32)	20	47(10)	26(3)	84.2%	Brazil	3	45	Women with FMS (ACR 1990), aged 18-60; sedentary; newly diagnosed, untreated	Supervised walking at a target HR set one workload below the anaerobic threshold.	NR
	flexibility/stretching (28)	20	44(11)	28(6)	73.7%	Brazil	3	45	Women with FMMS (ACR 1990), aged 18-60; sedentary; newly diagnosed, untreated	Whole-body stretching; 30-s holds; designed to avoid increasing HR.	NR
Wang et al. 2010 [23]	MBE (33)	12	49.7(11.8)	33.9(8.9)	85%	USA	2	60	Adults with FS (ACR 1990), aged ≥ 21	Yang-style Tai Chi with breathing and relaxation; master-led sessions plus home practice.	NR
	CG (33)	12	50.5(10.5)	31.5(7.4)	85%	USA	2	60	Adults with FMS (ACR 1990), aged ≥ 21	Health education plus supervised and home stretching; active control.	NR
Yavuz et al. 2023 [24]	AE (26)	6	38.12(9.02)	27.40(5.64)	83.9%	Turkiye	3	60	Women with FMS (ACR 2011), aged 18-60; PLI ≥17; SIQR ≥21	Supervised treadmill walking; 55-60% maximal HR progressing to moderate intensity; Borg-monitored.	NR
	Balance (25)	6	41.00(5.81)	26.43(3.77)	80.6%	Turkiye	3	60	Women with FMS (ACR 2011), aged 18-60; PLI ≥17; SIQR ≥21	Supervised balance/proprioception training; fixed to unstable surfaces and eyes open to closed progression.	NR

Note: Values are presented as mean (SD) unless otherwise specified. BMI is reported in kg/m². Exercise frequency refers to sessions per week, and duration refers to minutes per session. No-exercise controls were coded as 0 MET-min/week. Adherence is expressed as the reported completion or attendance percentage. Detailed exercise protocols were abbreviated while retaining the core exercise components, supervision, intensity, and progression. NR indicates not reported.

Abbreviations: ACR, American College of Rheumatology; AE, aerobic exercise; AQE, aquatic exercise; BMI, body mass index; CET, combined exercise training; CG, control group; CPET, cardiopulmonary exercise testing; FMS, fibromyalgia syndrome; HIIT, high-intensity interval training; HR, heart rate; Hz, hertz; ICD-10-CM, International Classification of Diseases, 10th Revision, Clinical Modification; MBE, mind-body exercise; MET, metabolic equivalent; NR, not reported; PLI, pain location inventory; PYD, pyridoxine; RPE, rating of perceived exertion; RT, resistance training; SBE, short-bout exercise; SD, standard deviation; SIQR, Symptom Impact Questionnaire-Revised; VAT, ventilatory anaerobic threshold; VO2max, maximal oxygen uptake; WBVT, whole-body vibration training; 1RM, one-repetition maximum.

4. Risk of bias

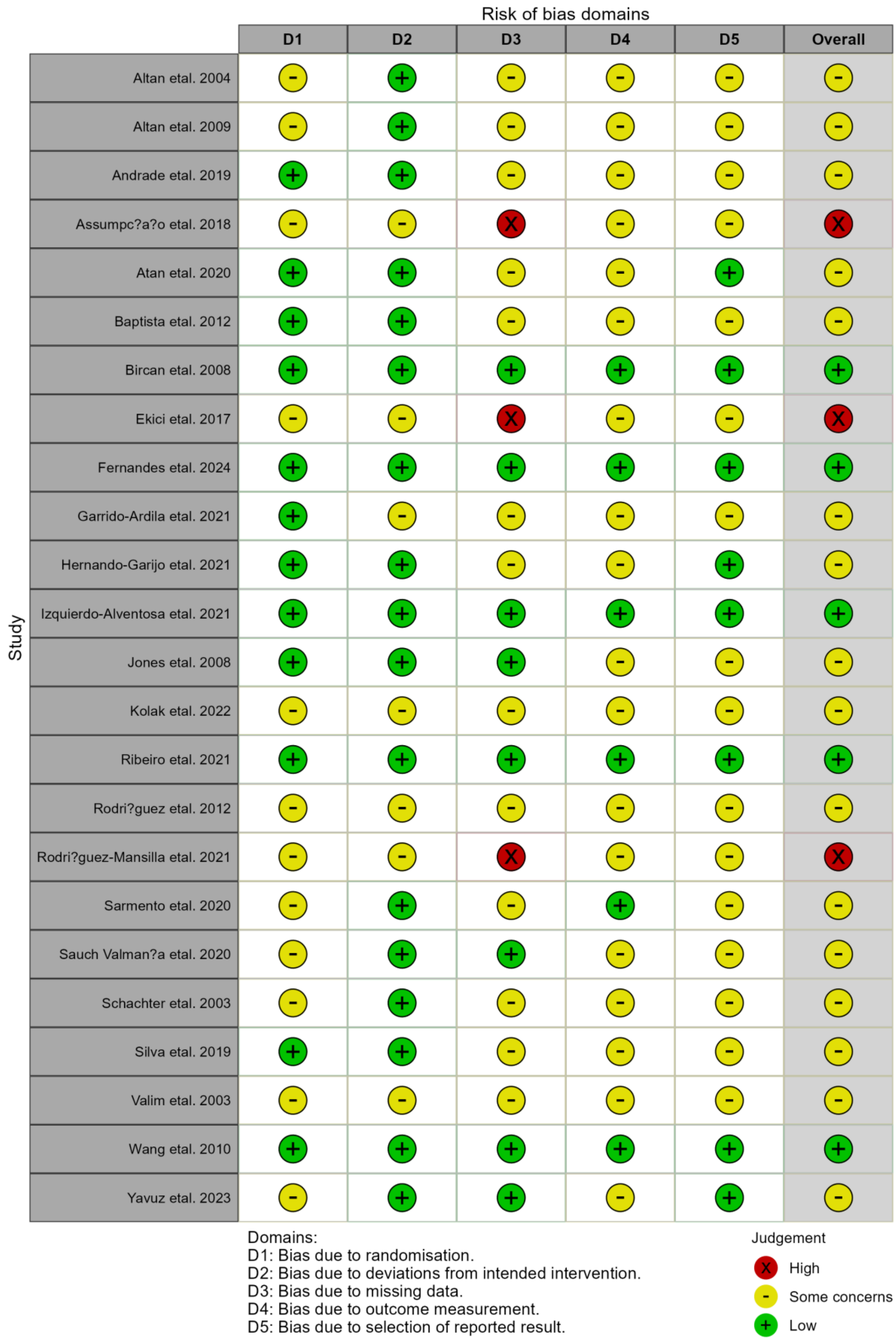

Figure S1. Risk-of-bias judgments for individual studies.

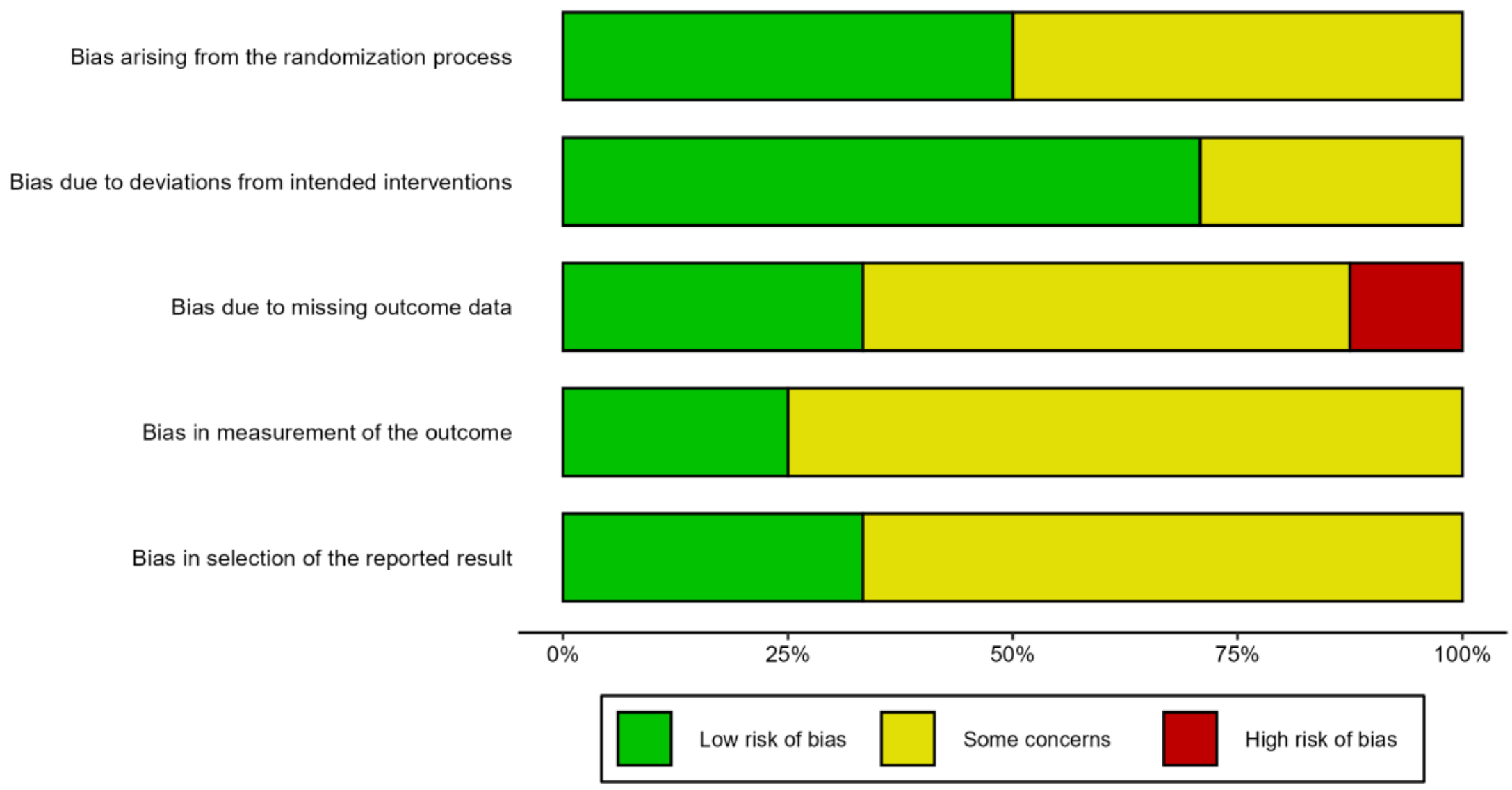

Figure S2. Summary of risk-of-bias judgments.

5. Dose-response network meta-analysis

5.1 Network connectivity

Connectivity is a fundamental assumption in network meta-analysis (NMA). In our study, the dose-based network allowed connections across both intervention modalities and dose levels, with no evidence of network disconnection observed.

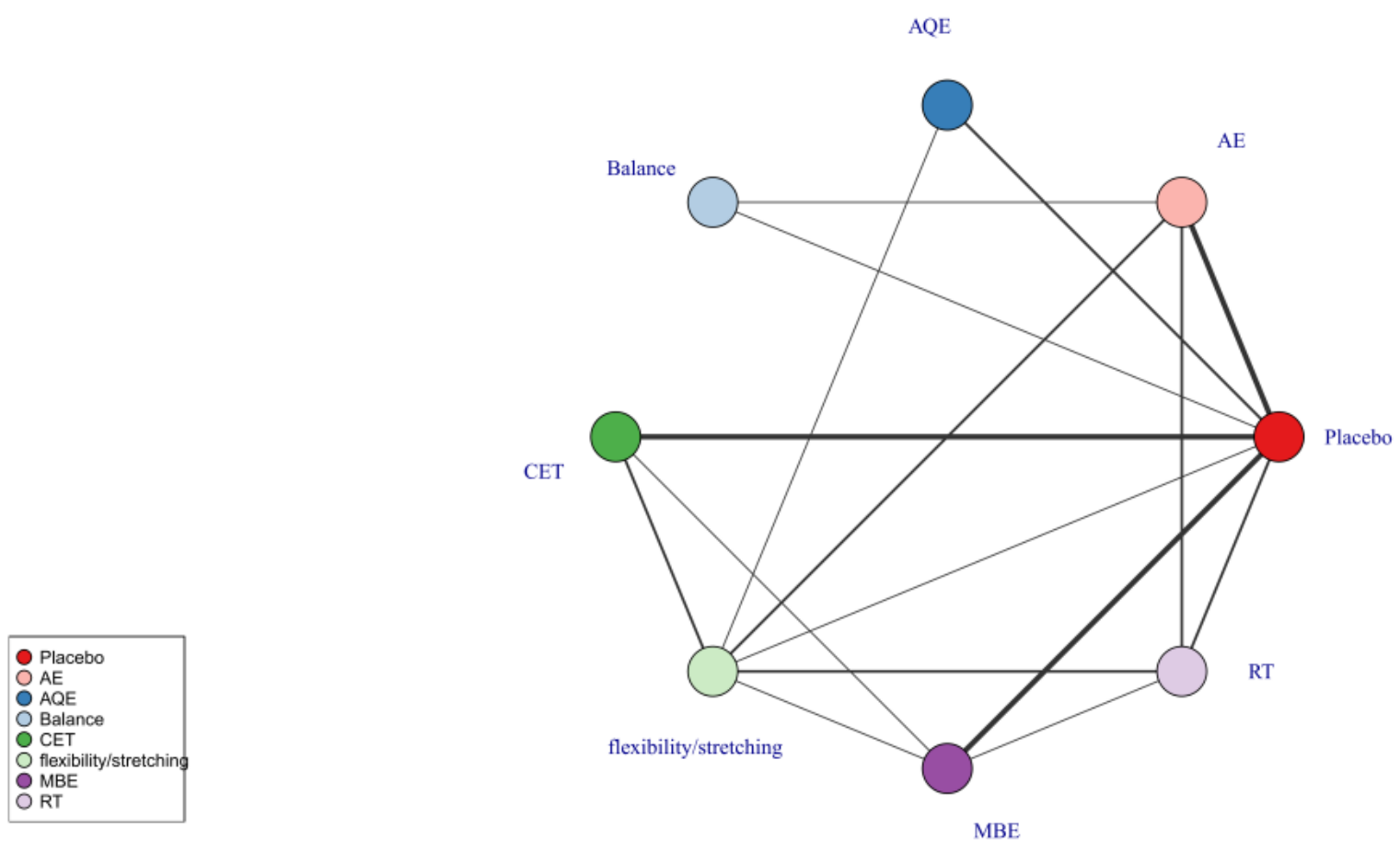

Figure S3. Exercise-level network.

Line thickness represents the number of studies available for comparison.

AE: Aerobic Exercise; AQE: Aquatic Exercise; Balance: Balance/Proprioceptive Training; CET: Combined Exercise Training; flexibility/stretching: Flexibility/Stretching Training; MBE: Mind-Body Exercise; RT: Resistance Training.

5.2 Consistency

Global consistency was assessed by comparing the fit of the consistency model with that of the unrelated mean effects (UME) model using the deviance information criterion, residual deviance, and between-study standard deviation (Table S3).

Table S3. Comparison of consistency and UME model fit.

Model	PD	Deviance	Residual deviance	DIC	SD
Consistent	47.8	66.235	53.148	114.0	1.062
UME	50.6	67.569	54.481	118.4	1.124

Note: UME = unrelated mean effects model; DIC = deviance information criterion; PD = effective number of parameters; SD = standard deviation

5.3 Transitivity

NMAs rely on the assumption of transitivity, which means that estimates of treatment effects derived from direct and indirect comparisons are consistent, accounting for the usual variability expected under a random-effects model. This assumption is analogous to the concept of heterogeneity in a standard meta-analysis. Following previous recommendations, we evaluated potential anomalies at a more granular level, specifically at the treatment level. The span of the network was assessed using the MBNMA node-splitting method, which separates the contribution of each treatment contrast into direct and indirect evidence and compares them. Consistent effects between these sources indicate good transitivity (p>0.05). Results of this assessment are presented in Table S4.

Table S4. Node-splitting analysis of consistency.

Comparison	p-value	Median	2.5%	97.5%
MBE_800 vs MBE_700	**0.008**			
-> direct		-2.593	-5.111	-0.108
-> indirect		-0.031	-0.093	-0.002
-> MBNMA		-0.032	-0.090	-0.002
RT_450 vs MBE_150	**0.520**			
-> direct		-1.428	-3.611	0.822
-> indirect		-0.298	-1.853	1.422
-> MBNMA		-0.713	-1.999	0.689
MBE_550 vs flexibility/stretching_450	**0.109**			
-> direct		-1.675	-4.076	0.660
-> indirect		1.617	-0.083	3.327
-> MBNMA		1.653	0.337	2.959
flexibility/stretching_200 vs CET_400	**0.320**			
-> direct		1.161	-1.341	3.541
-> indirect		-0.628	-2.005	0.720
-> MBNMA		-0.762	-1.867	0.408
flexibility/stretching_200 vs CET_300	**0.309**			
-> direct		1.228	-1.168	3.602
-> indirect		-0.582	-1.913	0.712
-> MBNMA		-0.707	-1.783	0.421
flexibility/stretching_300 vs AQE_350	**0.474**			
-> direct		1.395	-0.931	3.772
-> indirect		-0.121	-2.095	1.793
-> MBNMA		-0.637	-2.071	0.809
RT_400 vs AE_550	**0.731**			

-> direct		0.087	-2.319	2.417
-> indirect		-0.305	-1.725	1.291
-> MBNMA		-0.215	-1.490	1.163
flexibility/stretching_150 vs AE_550	**0.102**			
-> direct		1.512	-1.090	4.005
-> indirect		-1.685	-2.961	-0.352
-> MBNMA		-1.645	-2.734	-0.527
Balance_350 vs AE_550	**0.424**			
-> direct		-0.297	-2.480	1.865
-> indirect		1.571	-0.909	4.224
-> MBNMA		0.506	-1.234	2.189
RT_550 vs AE_500	**0.270**			
-> direct		1.294	-1.202	3.919
-> indirect		-0.871	-2.306	0.607
-> MBNMA		-0.310	-1.608	1.081
flexibility/stretching_300 vs AE_400	**0.137**			
-> direct		1.325	-1.096	3.700
-> indirect		-1.470	-2.966	-0.040
-> MBNMA		-1.469	-2.657	-0.281
RT_250 vs Placebo_0	**0.394**			
-> direct		-0.945	-3.430	1.417
-> indirect		-2.421	-3.577	-1.188
-> MBNMA		-2.025	-3.144	-0.813
RT_100 vs Placebo_0	**0.072**			
-> direct		-3.804	-5.828	-1.750
-> indirect		-1.089	-2.114	-0.229
-> MBNMA		-1.655	-2.809	-0.570
MBE_550 vs Placebo_0	**0.329**			
-> direct		-2.896	-4.457	-1.234
-> indirect		-1.496	-2.756	-0.159
-> MBNMA		-2.049	-3.078	-0.959
MBE_400 vs Placebo_0	**0.706**			
-> direct		-1.901	-4.063	0.304
-> indirect		-1.944	-3.090	-0.758
-> MBNMA		-1.940	-2.873	-0.921

MBE_300 vs Placebo_0	**0.427**			
-> direct		-0.961	-2.937	1.077
-> indirect		-2.029	-3.040	-0.980
-> MBNMA		-1.822	-2.710	-0.875
CET_550 vs Placebo_0	**0.627**			
-> direct		-0.730	-3.174	1.596
-> indirect		-1.250	-2.488	-0.069
-> MBNMA		-1.152	-2.175	-0.100
CET_500 vs Placebo_0	**0.516**			
-> direct		-0.373	-2.785	2.001
-> indirect		-1.317	-2.555	-0.153
-> MBNMA		-1.139	-2.147	-0.099
CET_200 vs Placebo_0	**0.670**			
-> direct		-1.094	-3.334	1.110
-> indirect		-0.933	-1.989	0.123
-> MBNMA		-0.959	-1.876	-0.087
Balance_300 vs Placebo_0	**0.432**			
-> direct		-0.559	-2.867	1.719
-> indirect		-2.334	-4.818	0.059
-> MBNMA		-1.405	-3.101	0.191
AQE_600 vs Placebo_0	**0.500**			
-> direct		-0.268	-2.612	2.103
-> indirect		-1.661	-3.488	0.209
-> MBNMA		-1.067	-2.496	0.275
AQE_500 vs Placebo_0	**0.813**			
-> direct		-1.350	-3.650	1.081
-> indirect		-0.962	-2.740	0.869
-> MBNMA		-1.045	-2.452	0.266
AE_1400 vs Placebo_0	**0.544**			
-> direct		-2.981	-5.209	-0.879
-> indirect		-2.043	-3.281	-0.852
-> MBNMA		-2.157	-3.326	-1.035
AE_1200 vs Placebo_0	**0.395**			
-> direct		-3.174	-5.533	-0.910
-> indirect		-1.770	-3.115	-0.552
-> MBNMA		-2.131	-3.284	-1.029

AE_400 vs Placebo_0	**0.564**			
-> direct		-1.386	-3.858	1.118
-> indirect		-1.892	-2.984	-0.883
-> MBNMA		-1.844	-2.782	-0.876
AE_200 vs Placebo_0	**0.206**			
-> direct		0.004	-2.203	2.150
-> indirect		-1.783	-2.785	-0.967
-> MBNMA		-1.525	-2.454	-0.744

Note: Treatment labels are followed by weekly dose in MET-min/week. AE: Aerobic Exercise; AQE: Aquatic Exercise; Balance: Balance/Proprioceptive Training; CET: Combined Exercise Training; flexibility/stretching: Flexibility/Stretching Training; MBE: Mind-Body Exercise; RT: Resistance Training.

To assess the assumption of transitivity, we examined the distribution of key baseline characteristics, including age, intervention duration, BMI, and baseline pain severity, across different intervention groups using boxplots. The Kruskal–Wallis H test, a non-parametric method for comparing continuous variables across multiple independent groups, was used to assess whether significant differences existed between intervention groups. The corresponding P values are presented below, with P > 0.05 indicating no statistically significant differences, suggesting that these baseline characteristics were comparable across the network and supporting the transitivity assumption.

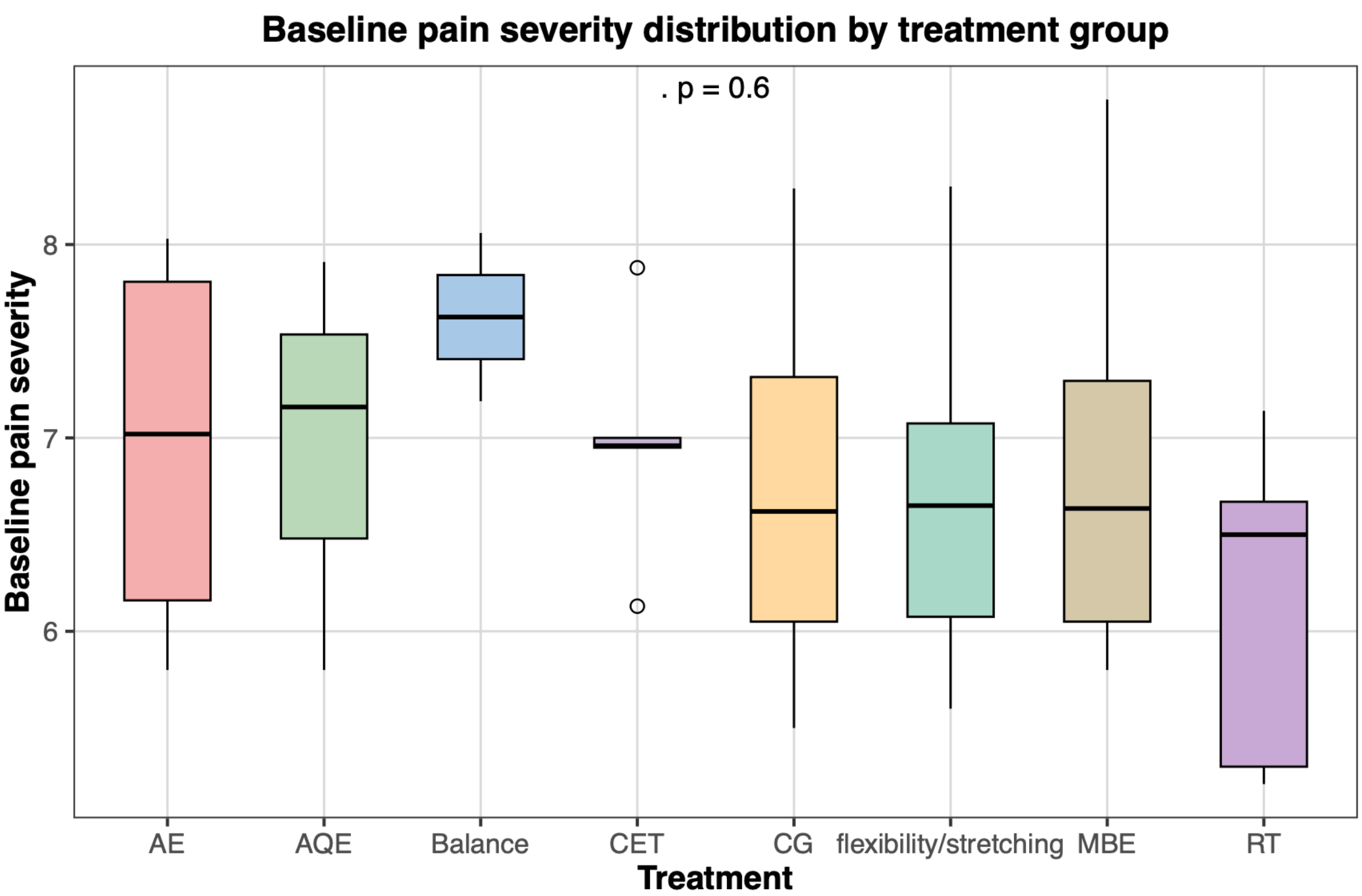

Figure S4. Distribution of baseline pain severity across intervention groups for assessment of transitivity

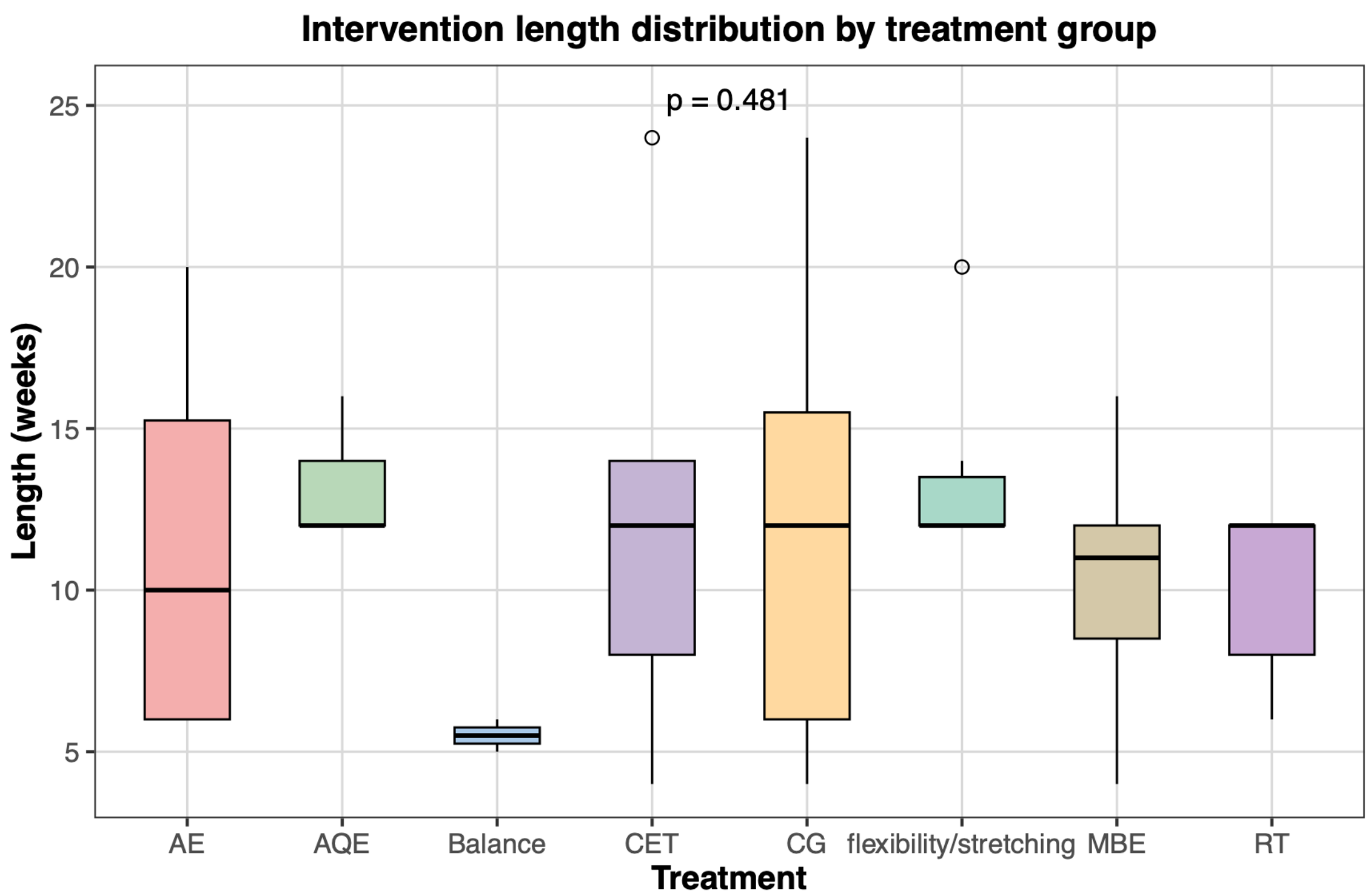

Figure S5. Distribution of intervention length across intervention groups for assessment of transitivity

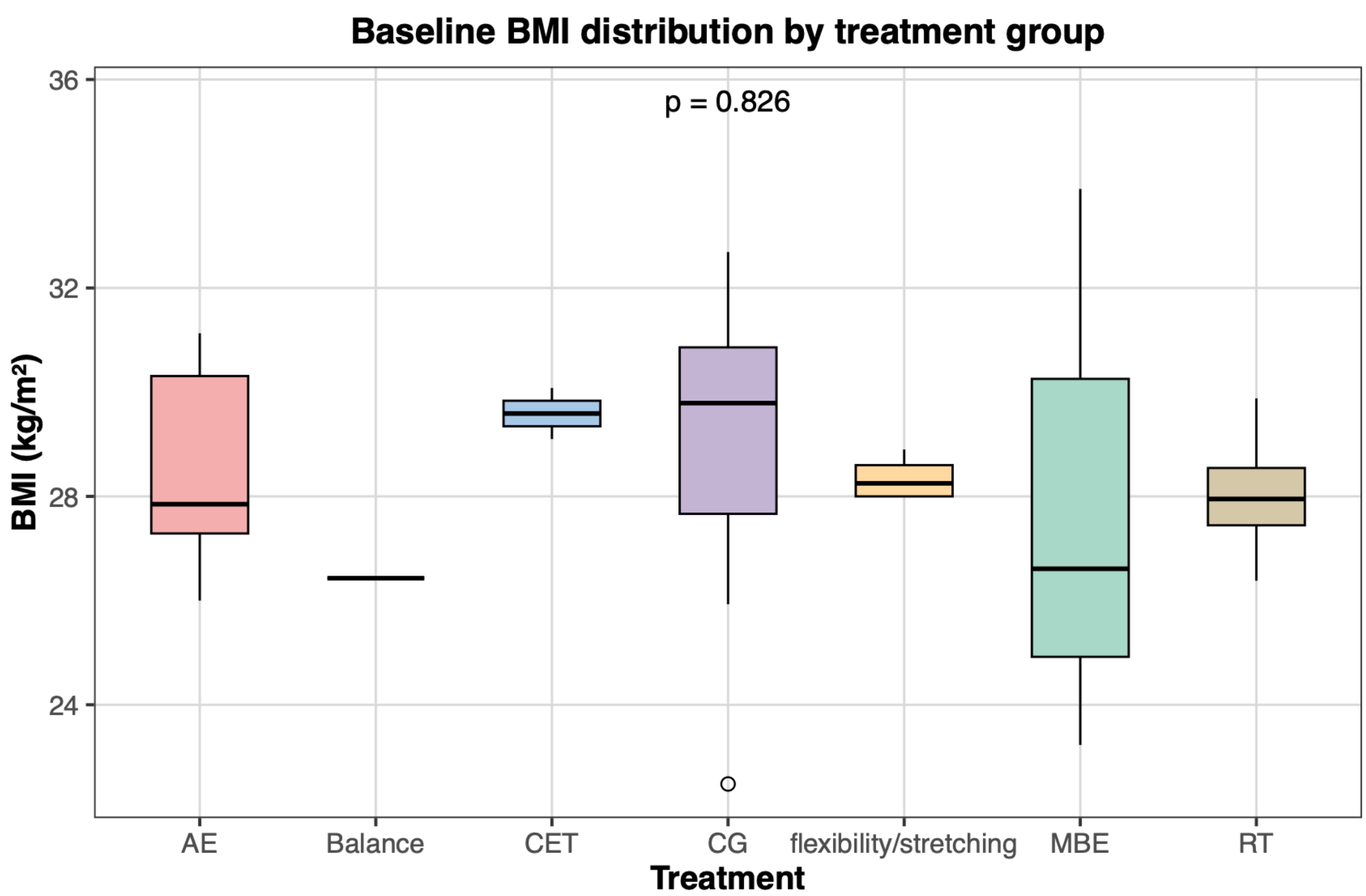

Figure S6. Distribution of BMI across intervention groups for assessment of transitivity

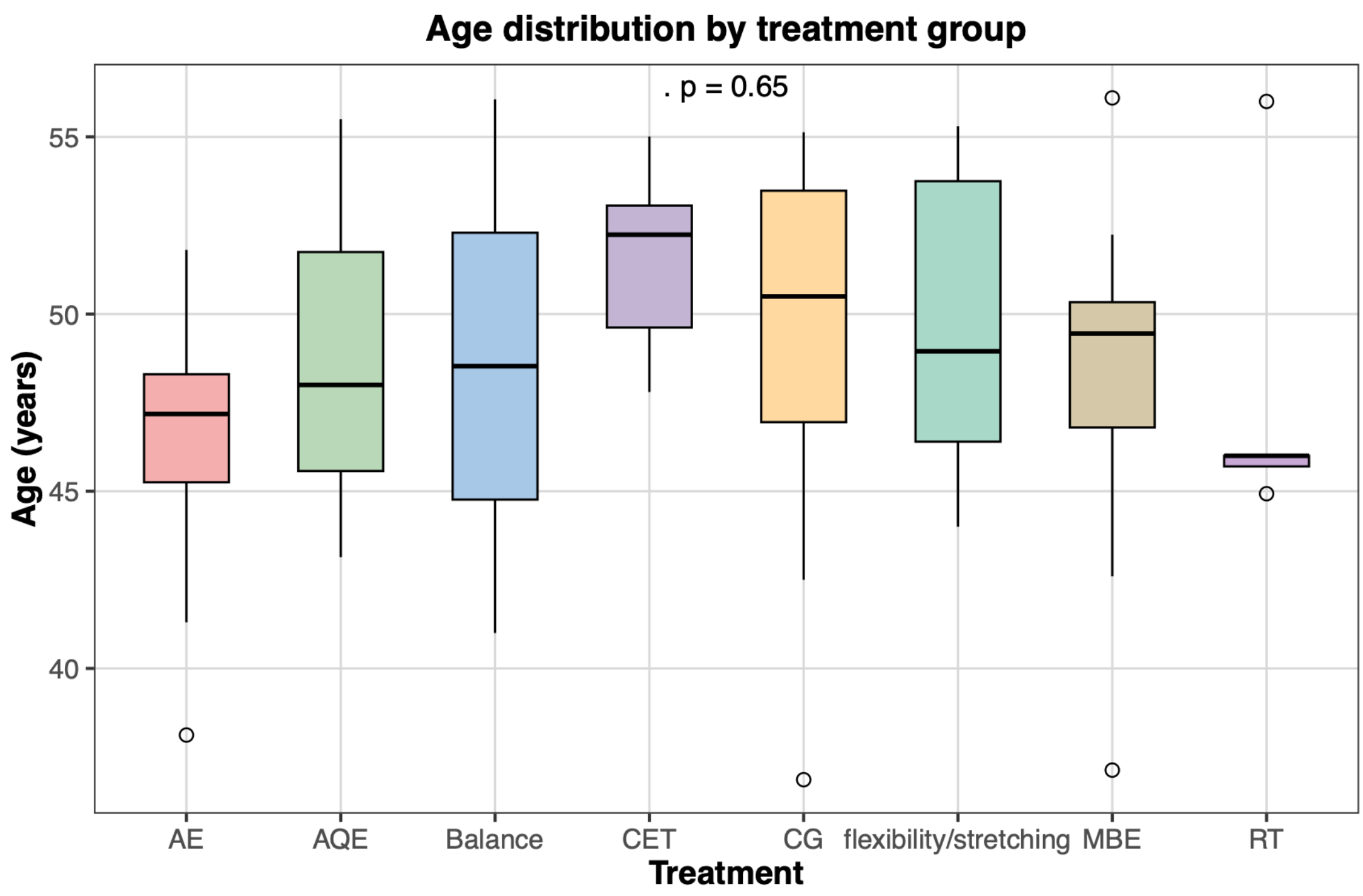

Figure S7. Distribution of age across intervention groups for assessment of transitivity

5.4 Dose-response model selection

For model selection, we adopted a quadratic function model. In accordance with the default settings in MBNMAdose, we applied vague (non-informative) prior distributions to minimize bias and allow the data to drive the results, thereby enabling the model to adapt flexibly to the underlying data structure. This approach is designed to capture a specific form of non-linear dose–response relationship and to identify an optimal dose under the assumption of a quadratic association. Below, we present a comparison of parameter estimates across different models (Table S5). In general, lower DIC indicate a better model fit [25]. As the quadratic function model exhibited the lowest DIC value, we selected it for our analysis.

Table S5. Model fit comparison.

Model	DIC	SD	Deviance	Residual deviance	pD
Emax (random treatment effects)	113.8	1.006	67.413	54.325	47.2
Restricted cubic spline (random treatment effects; 3 knots)	119.8	0.888	66.524	53.436	52.9
Log Linear (random treatment effect)	114.6	1.024	67.214	54.126	47.6

Quadratic function (random treatment effects)	113.3	1.082	66.647	53.560	48.3

Note: DIC: deviance information criterion; pD: effective number of parameters; SD: standard deviation.

We also validated the fit of the quadratic function model to the data. Figures S4 and S5 present the results of the "Split" network meta-analysis at different doses of overall and various exercise intervention on pain. This step aimed to explore the dose–response pattern and to assess which functional form best fits the data, thereby informing the selection of a model for the subsequent MBNMA. The model that best matches the pattern shown in Figure S4 and S5 should be selected accordingly.

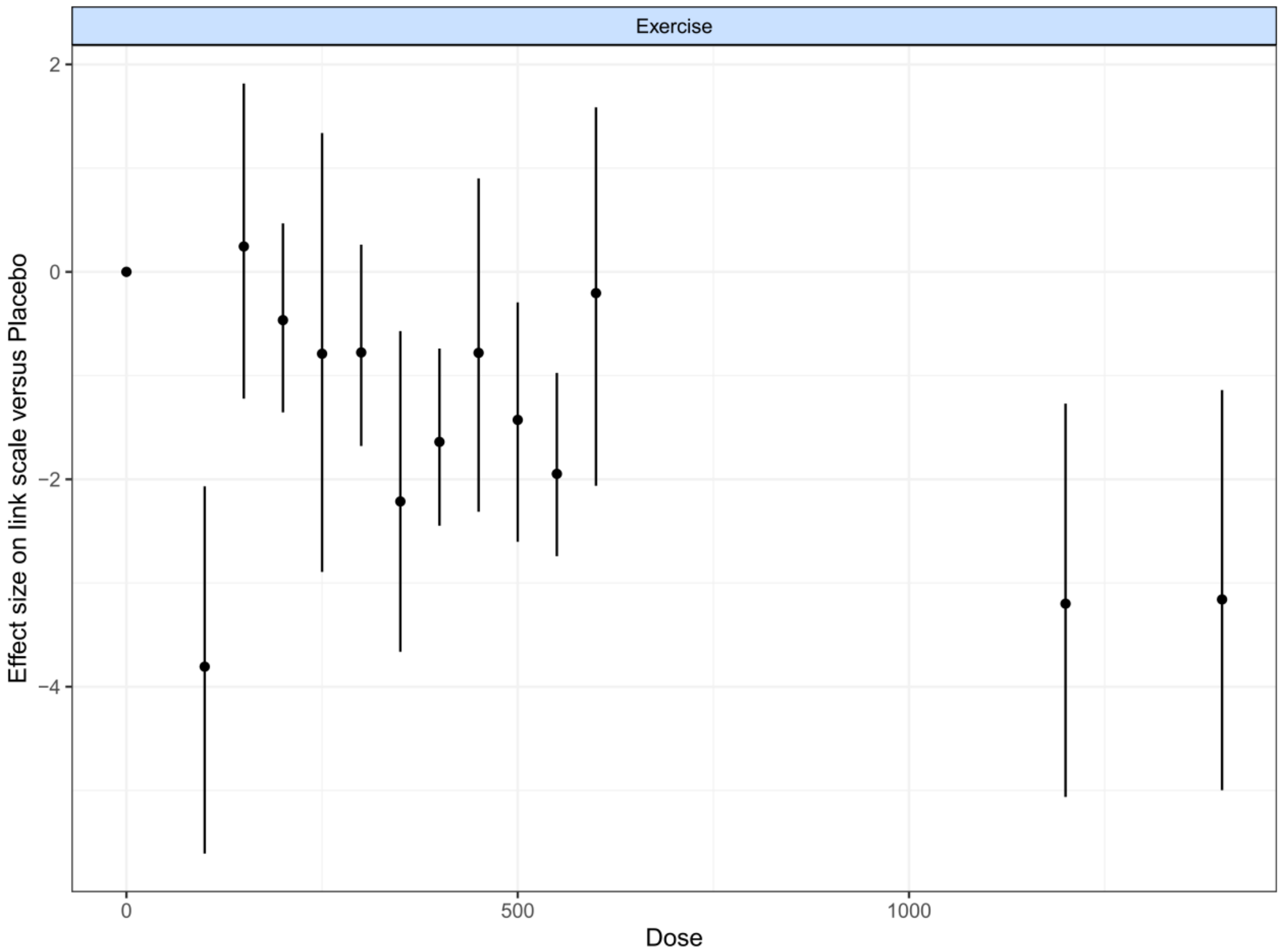

Figure S8. Node-splitting network meta-analysis of exercise interventions for pain intensity in adults women with FMS.

The black dots represent the pooled effect size from the meta-analysis at that dose, and the black vertical lines represent the range of the 95% CrI.

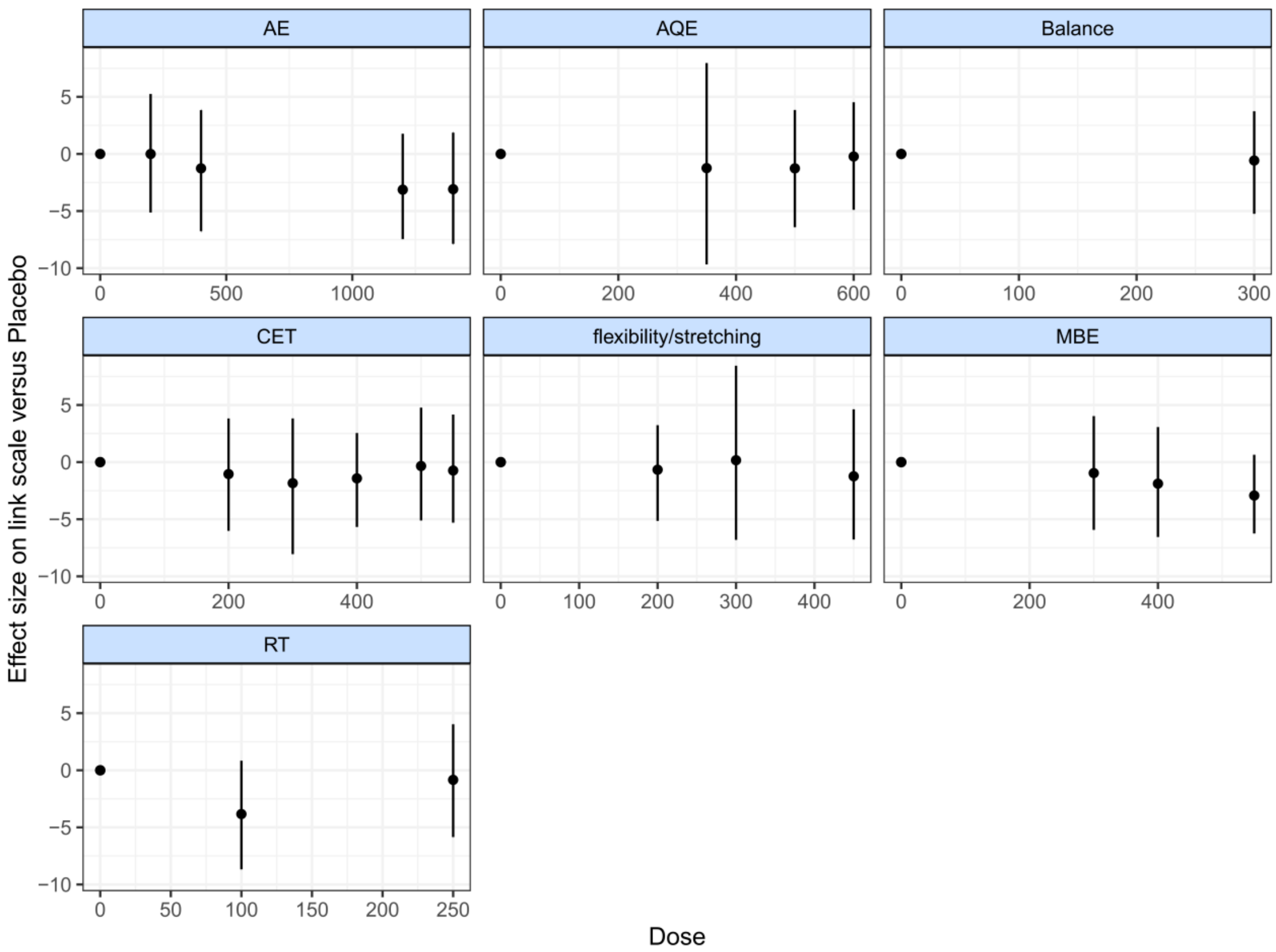

Figure S9. Node-splitting network meta-analysis of different exercise modalities for pain intensity in adults women with FMS.

The black dots represent the pooled effect size from the meta-analysis at that dose, and the black vertical lines represent the range of the 95% CrI. AE: Aerobic Exercise; AQE: Aquatic Exercise; Balance: Balance/Proprioceptive Training; CET: Combined Exercise Training; flexibility/stretching: Flexibility/Stretching Training; MBE: Mind-Body Exercise; RT: Resistance Training.

We observed that the trend of the model curve is generally consistent with the data points from the "Split" network meta-analysis, suggesting a good fit between the model and the data.

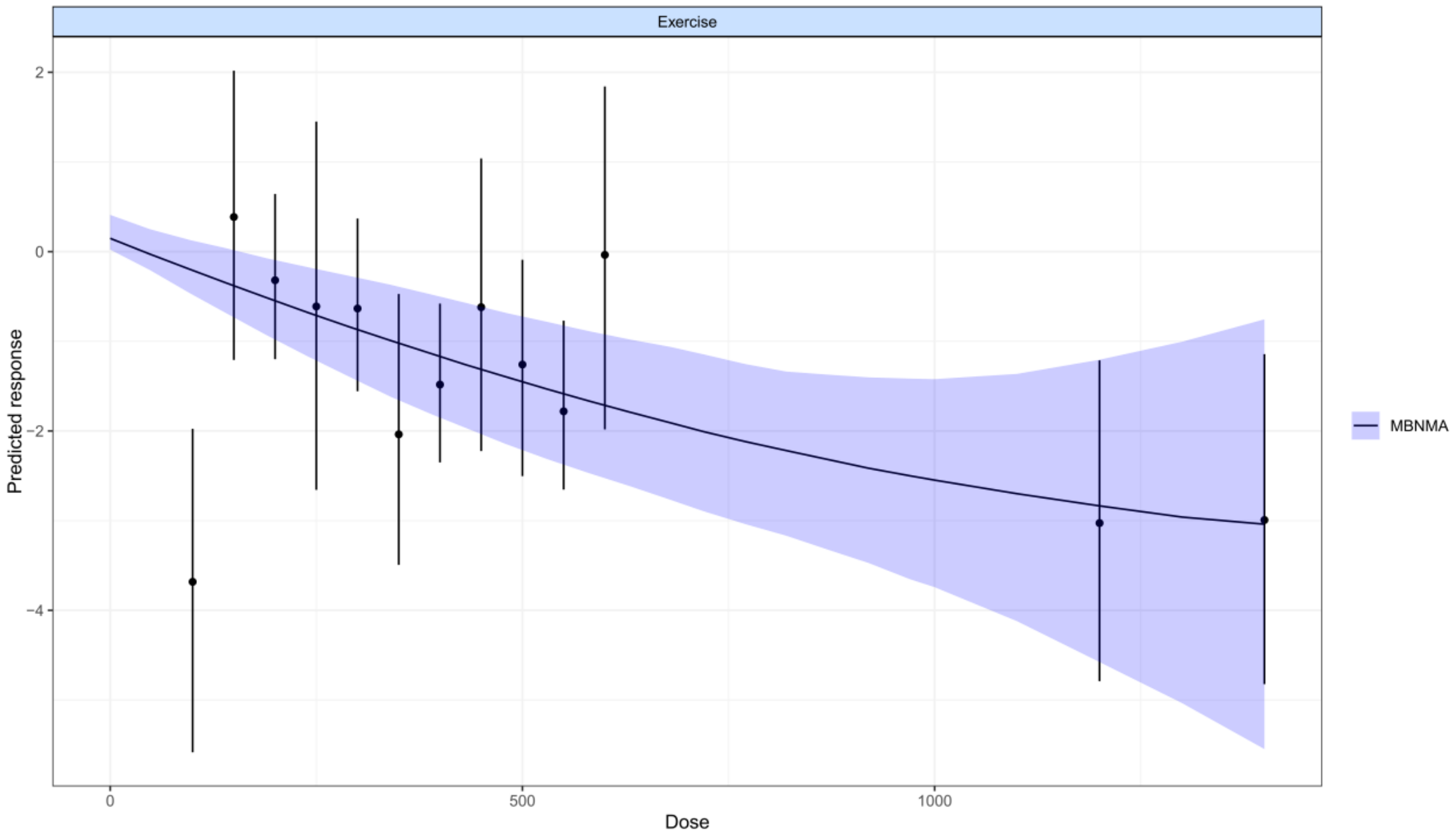

Figure S10. Dose-response relationship between overall exercise dose and pain intensity in adults with FMS (quadratic function).

The black dots represent the pooled effect size from the meta-analysis at that dose, while the blue region represents the 95% CrI range.

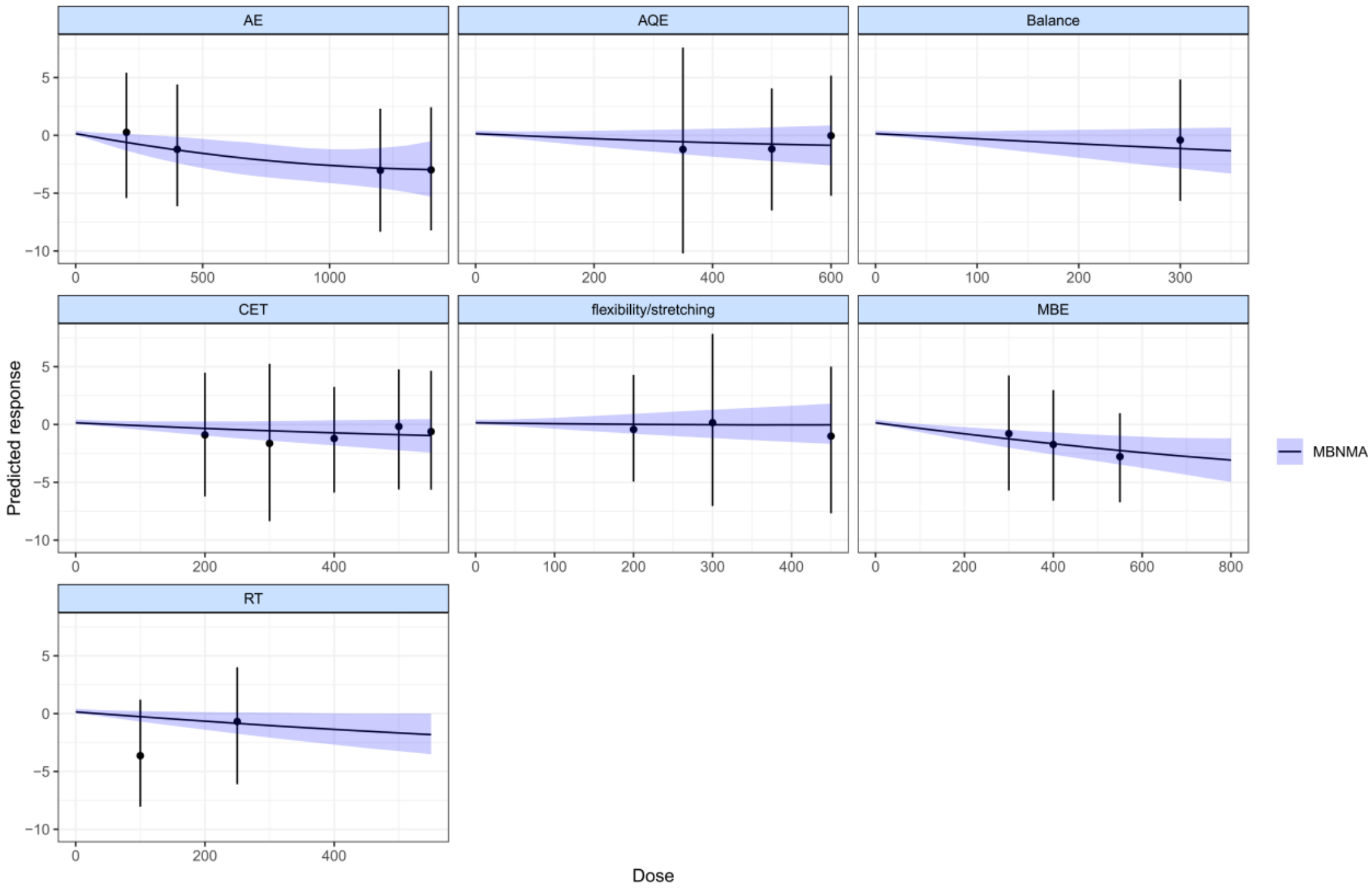

Figure S11. Dose-response relationships between different exercise modalities and pain intensity in adults women with FMS (quadratic function).

The black dots represent the pooled effect size from the meta-analysis at that dose, while the blue region represents the 95% CrI range. AE: Aerobic Exercise; AQE: Aquatic Exercise; Balance: Balance/Proprioceptive Training; CET: Combined Exercise Training; Flexibility/Stretching: Flexibility/Stretching Training; MBE: Mind-Body Exercise; RT: Resistance Training.

5.5 Dose-response relationships

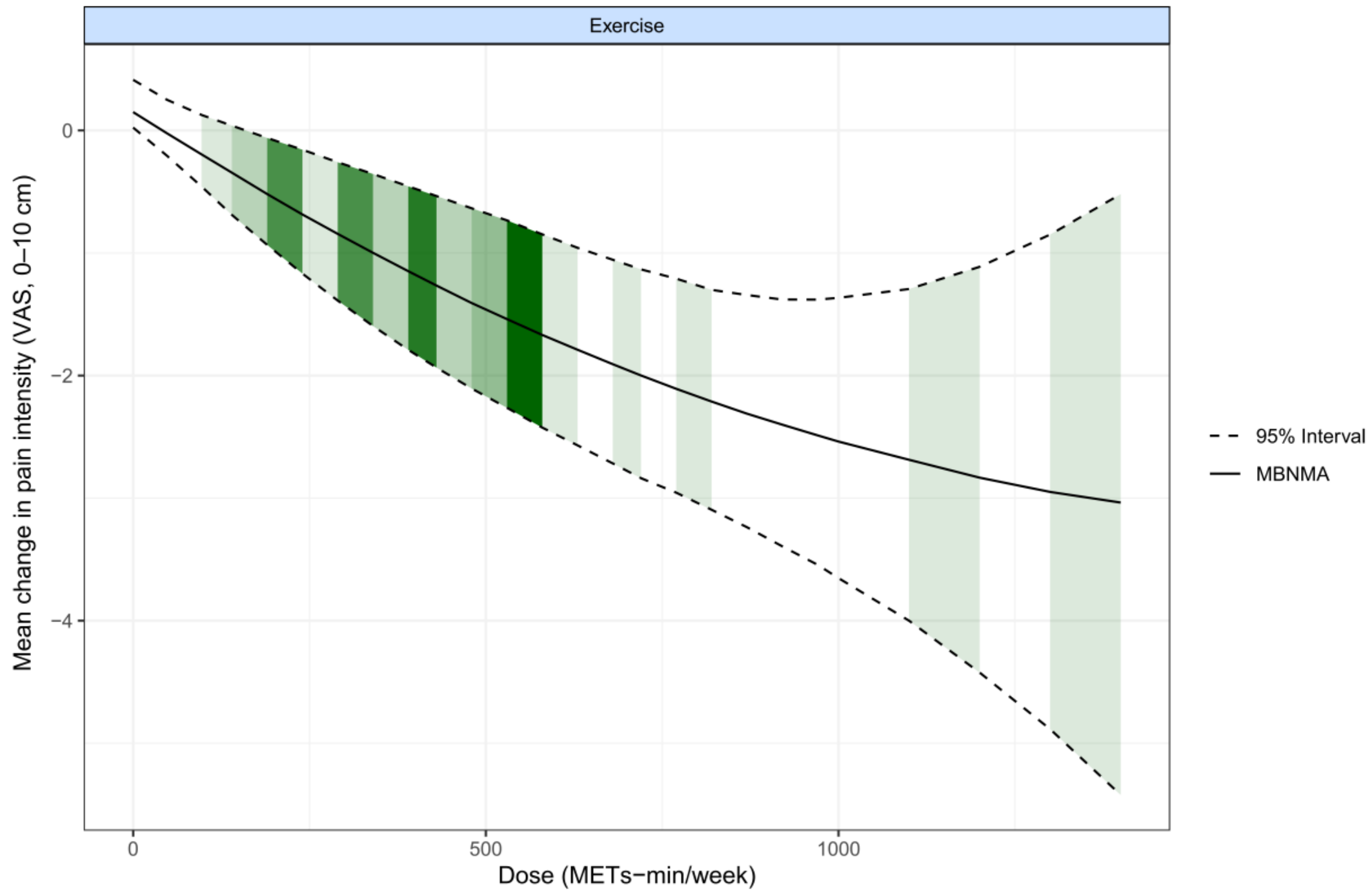

Figure S12. Dose-response relationship between overall exercise dose and pain intensity in adults women with FMS. (the green area represents the original study dataset; the darker the color, the larger the amount of data)

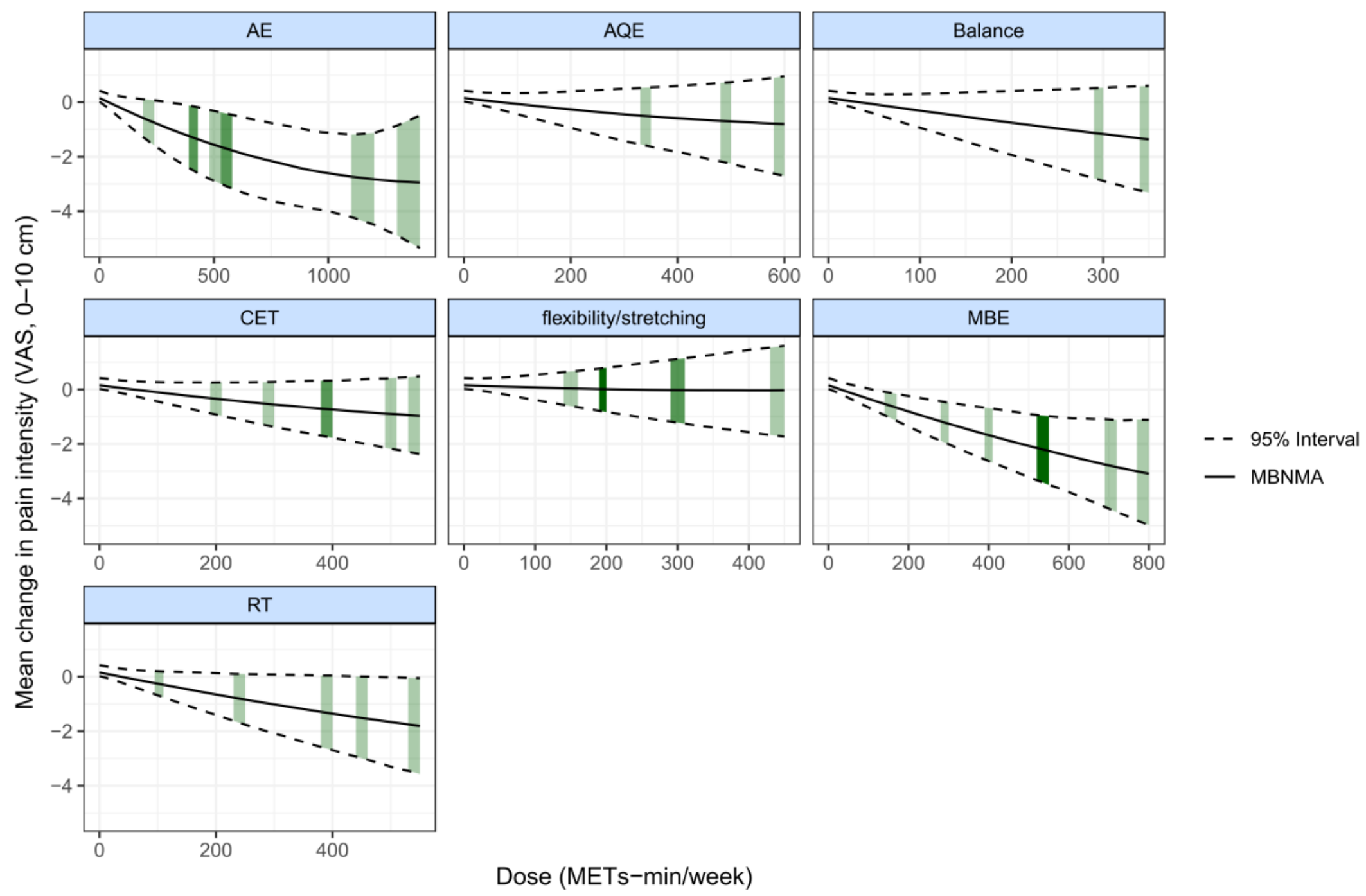

Figure S13. Dose-response relationships between different exercise modalities and pain intensity in adults with FMS.

(the green area represents the original study dataset; the darker the color, the larger the amount of data)

5.6 Sensitivity analysis

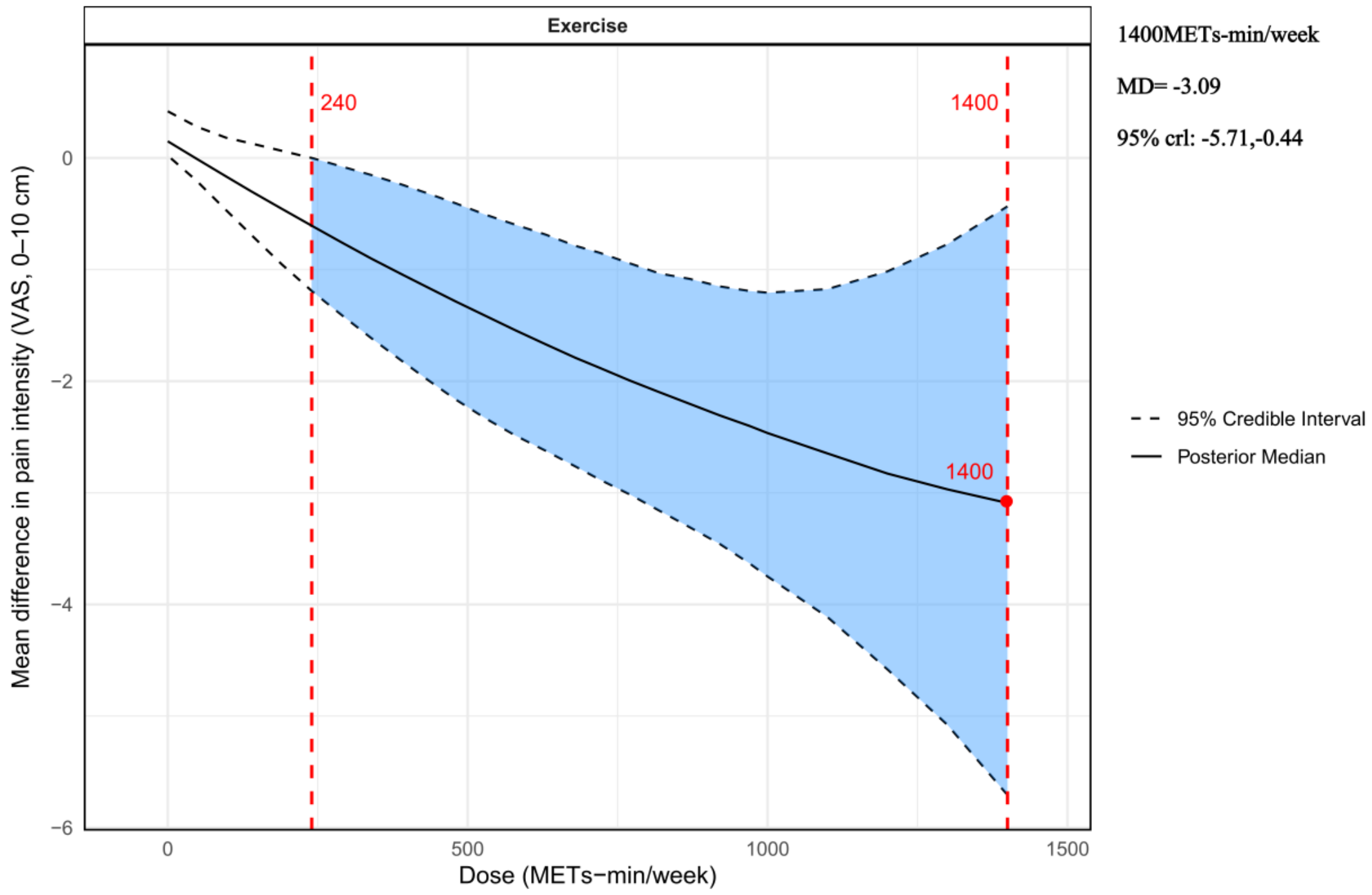

Figure S14. Dose-response relationship between overall exercise and pain intensity in the sensitivity analysis excluding studies at high risk of bias.

The red dashed vertical lines indicate the model-identified effective dose range from 240 to 1400 MET-min/week. The red dot marks the optimal dose at 1400 MET-min/week, corresponding to a mean difference of -3.09 points on the 0-10 cm visual analogue scale (95% CrI, -5.71, -0.44). Negative values indicate a reduction in pain intensity.

5.7 Relative effectiveness ranking

Table S6. Relative effectiveness rankings across intervention modes and doses.

Rank	Treatment	Mean	Median	2.50%	97.50%
1	MBE_800	10.04833333	2	1	66.025
2	MBE_770	11.07633333	4	2	62.025
3	MBE_740	12.251	6	3	60
4	MBE_720	13.39933333	7	4	58
5	AE_1300	15.657	8	2	80
6	AE_1400	18.955	8	1	116
7	AE_1200	14.125	9	2	62
8	MBE_690	14.79266667	9	5	56
9	AE_1100	13.88533333	11	1	51
10	MBE_660	16.36733333	11	6	56
11	AE_1000	14.81333333	12	2	44
12	MBE_630	18.10166667	13	7	57.025
13	AE_970	15.79833333	14	2	46
14	AE_920	17.299	15	2	48
15	MBE_610	19.68266667	15	8	59
16	AE_870	19.23166667	17	2	51
17	MBE_580	21.739	17	9	61
18	AE_820	21.49333333	19	3	56.025
19	MBE_550	24.032	20	10	64
20	AE_770	24.165	22	4	64
21	MBE_520	26.57033333	23	11	66.025
22	AE_720	27.185	24	6	70
23	MBE_500	28.74266667	25	12	69
24	AE_680	30.133	27	8	75
25	MBE_470	31.747	28	13	73
26	RT_550	36.61933333	29	1	124
27	AE_630	33.90666667	30	10	81.025
28	RT_530	38.829	31	2	122.025
29	MBE_440	35.04966667	32	14	77
30	AE_580	38.183	34	12	90
31	RT_510	41.19033333	34	3	127
32	MBE_410	38.71666667	36	15	81
33	RT_490	43.65233333	36	4	127.025
34	AE_530	42.99	39	13.975	99
35	MBE_390	41.73466667	39	16	84
36	RT_470	46.26333333	39	7	127
37	RT_460	48.08866667	41	10	128.025
38	AE_480	48.47866667	44	16	107.025
39	MBE_360	46.17266667	44	19	89

Rank	Treatment	Mean	Median	2.50%	97.50%
40	RT_440	50.88633333	44	13	130
41	Balance_350	58.93933333	46	1	200
42	RT_420	53.762	47	16	132
43	Balance_340	60.99	48.5	2	199
44	MBE_330	51.14533333	49	23	95
45	RT_400	56.79933333	50	19	134
46	AE_430	54.81666667	51	17	116
47	Balance_330	63.13433333	51	5	198
48	RT_380	60.01766667	53	22	136.025
49	MBE_300	56.73033333	54	27	101
50	Balance_310	66.67633333	55	9	197
51	AE_390	60.80066667	57	21	122.025
52	RT_360	63.37566667	57	25	138
53	Balance_300	68.99	58	12	196
54	MBE_280	61.22366667	59	30	105.025
55	Balance_290	71.36333333	60	14.975	195
56	RT_340	66.885	61	27.975	140.025
57	Balance_280	73.808	64	17	194
58	AE_340	69.02533333	65	29	130
59	RT_320	70.60333333	65	31	142.025
60	CET_550	76.54066667	66	14	198
61	Balance_270	76.32766667	67	19	193
62	MBE_250	68.19233333	67	36	112
63	CET_530	77.99366667	68	16	195
64	RT_300	74.47133333	69	34.975	144
65	Balance_250	80.66766667	71	24	192
66	CET_510	79.572	71	19	193
67	AQE_600	86.80166667	73	10	200
68	CET_490	81.328	74	21	190
69	RT_280	78.69866667	74	38	146
70	AE_290	78.70533333	75	36	139.025
71	AQE_580	87.702	75	12	199
72	Balance_240	83.40133333	75	27	191
73	MBE_220	76.20766667	75	40	120.025
74	CET_470	83.22133333	76	24	187
75	RT_270	81.402	76	41	148
76	AQE_560	88.71533333	77	15	198
77	Balance_230	86.204	78	30	190
78	CET_460	84.56966667	78	26	186
79	AQE_540	89.85766667	80	17	197
80	AQE_520	91.10566667	81	19	196

Rank	Treatment	Mean	Median	2.50%	97.50%
81	CET_440	86.71333333	81	29	182
82	RT_250	85.98633333	81	46	150
83	Balance_220	89.05866667	82	32	189
84	AQE_500	92.50866667	83	22	195
85	CET_420	88.99433333	84	32	180
86	MBE_190	85.38833333	84	47	129
87	Balance_210	92.00033333	85	36	188
88	AQE_480	94.068	86	25	194
89	AE_240	90.077	87	46	149
90	CET_400	91.44733333	87	35.975	177
91	RT_230	90.92066667	87	51	154
92	AQE_460	95.75133333	89	27.975	193
93	CET_380	94.029	90	39	174
94	AQE_430	98.30266667	92	31.975	192
95	Balance_190	97.282	92	42	187
96	MBE_170	92.60333333	92	53	136
97	CET_360	96.789	93	42	173
98	RT_210	96.19433333	93	55	156
99	AQE_410	100.3936667	95	35	191
100	Balance_180	100.5246667	96	46	186
101	CET_340	99.73633333	97	45	172
102	AQE_390	102.6036667	98	39	190
103	RT_190	101.8053333	99	60	159.025
104	Balance_170	103.9046667	100	50	185
105	AQE_370	105.0136667	101	42	189
106	CET_320	102.9016667	101	48.975	171
107	AE_190	103.5646667	102	60	158
108	AQE_350	107.5573333	104	45	188
109	Balance_160	107.4106667	104	53	184
110	CET_300	106.2183333	104	52.975	170
111	MBE_140	104.11	104.5	62	146
112	RT_170	107.7726667	106	64	160.025
113	AQE_330	110.287	107	49	187
114	CET_280	109.747	108	57	169
115	AQE_310	113.2213333	111	52	186
116	Balance_140	113.9683333	111	60	183
117	CET_270	112.0196667	111	59	169.025
118	RT_150	114.2653333	113	70	165
119	AQE_290	116.273	115	55	185
120	Balance_130	117.889	115	65	182
121	CET_250	115.8323333	115	63.975	170

Rank	Treatment	Mean	Median	2.50%	97.50%
122	MBE_110	117.177	117	71	158
123	AQE_270	119.51	119	60	184
124	CET_230	119.7983333	119	69	170
125	AE_140	119.756	120	76	167
126	Balance_120	121.9546667	120	70	181
127	RT_130	121.321	120	77	169
128	AQE_250	122.9313333	123	65	183
129	CET_210	123.9816667	124	74	172
130	Balance_110	126.184	125	76	180
131	AQE_230	126.581	127	71	182
132	CET_190	128.4436667	129	80	174
133	RT_110	128.9913333	129	85	174
134	AQE_210	130.437	132	77	181
135	Balance_97	131.6576667	132	82	181
136	MBE_83	130.7426667	132	82	169
137	CET_170	133.102	134	86	176
138	AQE_190	134.4536667	136	83	180
139	RT_95	135.422	136	90	177
140	AE_97	136.411	138	92.975	175
141	Balance_84	137.519	138	88	184
142	CET_150	137.9526667	139	91	179
143	AQE_170	138.688	141	89	179
144	Balance_72	143.2666667	144	94	187
145	CET_130	143.0733333	145	96	181
146	RT_76	143.816	146	97	182
147	AQE_140	145.0933333	147	96	182
148	CET_110	148.4483333	149	102	184
149	MBE_55	146.771	149	96	181.025
150	Balance_60	149.3466667	150	99	189
151	AQE_120	149.8543333	151	102	185
152	CET_95	152.927	154	107	187
153	RT_57	152.9633333	154	105	187
154	AQE_100	154.8973333	156	107	187
155	flexibility/stretching_370	148.6636667	156	51	195
156	flexibility/stretching_390	147.8653333	156	47.975	196
157	flexibility/stretching_400	147.4963333	156	45	197
158	Balance_48	155.7673333	157	106	192
159	flexibility/stretching_420	146.888	157	41	198
160	flexibility/stretching_430	146.6236667	157	38	199
161	flexibility/stretching_360	149.1303333	157.5	54	194
162	flexibility/stretching_340	150.1096667	158	58.975	193

Rank	Treatment	Mean	Median	2.50%	97.50%
163	flexibility/stretching_450	146.1553333	158	35.975	200
164	AE_48	159.0413333	159	113	189
165	CET_76	158.6986667	159	115	190
166	flexibility/stretching_310	151.8296667	159	66	191
167	flexibility/stretching_330	150.6793333	159	62	192
168	AQE_83	159.5603333	160	115	190
169	flexibility/stretching_290	153.106	160	71.975	190
170	flexibility/stretching_280	153.849	161	75	189
171	flexibility/stretching_260	155.2693333	163	81	188
172	RT_38	162.91	163	113	193
173	Balance_36	162.5776667	164	116	194

Note: The number that follows the exercise intervention indicates the weekly exercise dose (MET-min/week). AE: Aerobic Exercise; AQE: Aquatic Exercise; Balance: Balance/Proprioceptive Training; CET: Combined Exercise Training; flexibility/stretching: Flexibility/Stretching Training; MBE: Mind-Body Exercise; RT: Resistance Training.

6. Meta-regression analysis

Table S7. Dose-response network meta-regression.

Covariate	DIC	Residual Deviance	Shared beta (median and 95% CrI)	SD (median and 95% CrI)
Age (years)	115.7	51.5	-0.090 (-0.226, 0.072)	1.03 (0.62, 1.63)
BMI (kg/m^2)	60.4	26.0	-0.102 (-0.50, 0.314)	1.49 (0.75, 3.43)
Intervention length (weeks)	116.6	51.2	0.049 (-0.072, 0.179)	1.07 (0.67, 1.71)

Note: DIC: deviance information criterion; SD: standard deviation

7. Publication bias

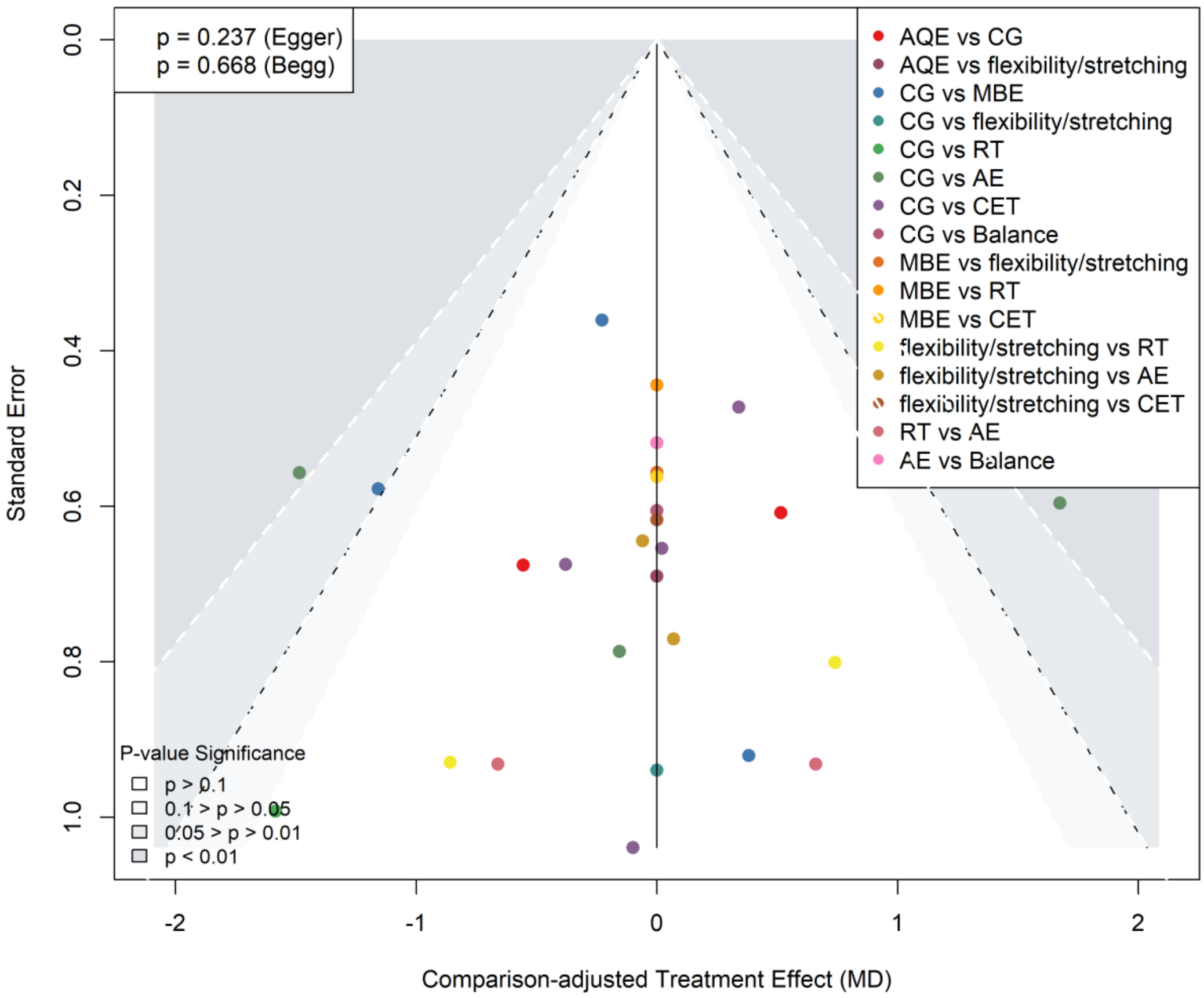

Figure S15. Comparison-adjusted funnel plot.

AE: Aerobic Exercise; AQE: Aquatic Exercise; Balance: Balance/Proprioceptive Training; CET: Combined Exercise Training; flexibility/stretching: Flexibility/Stretching Training; MBE: Mind-Body Exercise; RT: Resistance Training; CG: Control Group.

8. Confidence in the evidence using CINeMA

Following the CINeMA guidance (https://cinema.ispm.unibe.ch/), we assessed confidence in the evidence for the effects of different exercise modalities and exercise doses on pain intensity in adults women with fibromyalgia syndrome (FMS). Pain intensity was primarily assessed using visual analogue scales (VAS). Each network estimate was evaluated across six CINeMA domains.

8.1 Within-study bias

The risk of bias of each included randomized controlled trial was assessed using the Cochrane Risk of Bias 2 (RoB 2) tool and classified as low risk, some concerns, or high risk. CINeMA contribution matrices were used to determine the contribution of each study to each network estimate. Exercise interventions cannot generally be double-blinded; therefore, the absence of participant or therapist blinding was not considered sufficient to classify a study as having high risk of bias. Downgrading was based on the risk of bias of the studies contributing most substantially to each network estimate. No concerns were assigned when the evidence was predominantly contributed by studies at low risk of bias, some concerns were assigned when important contributions came from studies with some concerns, and major concerns were assigned when the estimate was substantially influenced by studies at high risk of bias.

8.2 Reporting bias

To minimize the risk of publication and selective reporting bias, we searched the reference lists of relevant systematic reviews and included studies. Reporting bias was assessed using comparison-adjusted funnel plots and Begg's and Egger's tests when a sufficient number of studies were available. Statistical test results were interpreted together with funnel-plot asymmetry, study size, the distribution of exercise modalities and doses, and evidence of selective reporting of pain outcomes or follow-up time points. The domain was not downgraded when no meaningful evidence of publication bias or selective outcome reporting was identified.

8.3 Indirectness

Indirectness was assessed according to the clinical and methodological relevance of the included studies to the review question. Eligible studies included adults women with FMS diagnosed according to recognized diagnostic criteria or by a qualified clinician. The interventions included aerobic exercise, resistance training, aquatic exercise, mind-body exercise, combined exercise training, balance or proprioceptive training, flexibility/stretching, and other eligible exercise modalities. Comparators included no-exercise controls, usual care, waiting-list controls, or active control interventions.

Pain intensity was evaluated using VAS-based measures. Differences in participant characteristics, diagnostic criteria, baseline pain severity, exercise modality, weekly exercise dose, intervention duration, frequency, session duration, intensity, supervision, and pain assessment procedures were considered when judging indirectness and the plausibility of the transitivity assumption.

8.4 Imprecision

Pain intensity was treated as a continuous outcome. The 95% credible intervals were examined to determine whether they crossed the null effect threshold of 0 and whether they included values compatible with both clinically important benefit and little or no benefit. Because a single minimal clinically important difference applicable to all FMS populations, VAS implementations, exercise modalities, and comparisons was not prespecified, the null threshold was used as the primary statistical decision threshold. This limitation was considered when interpreting the clinical importance of imprecise estimates.

8.5 Heterogeneity

Heterogeneity was assessed by considering the between-study variance and the extent to which credible and prediction intervals could lead to different clinical conclusions. Potential sources of heterogeneity included differences in baseline pain intensity, FMS diagnostic criteria, participant characteristics, exercise modality, weekly exercise dose, intervention duration, frequency, session duration, intensity, supervision, adherence, and outcome assessment. Comparisons or dose-specific estimates were downgraded when heterogeneity was sufficiently large to reduce confidence in the estimated effect or to produce materially different conclusions across studies.

8.6 Incoherence

Incoherence between direct and indirect evidence was assessed using appropriate local and global methods. Statistical significance was considered supporting evidence rather than the sole basis for judgment. The magnitude and uncertainty of disagreement between direct and indirect estimates were also examined, together with differences in participant characteristics, exercise modality, comparator type, dose category, and outcome measurement. Each network estimate was classified as having no concerns, some concerns, or major concerns for incoherence according to the extent to which disagreement between direct and indirect evidence could alter the interpretation of the estimated effect.

8.7 Overall CINeMA judgment

CINeMA evaluates confidence in each network estimate across six domains: within-study bias, reporting bias, indirectness, imprecision, heterogeneity, and incoherence. These domain-level judgments were integrated to classify the overall confidence in each comparison or dose-response estimate as high, moderate, low, or very low. Domains were considered jointly to avoid duplicate downgrading when the same underlying limitation affected more than one domain. Overall confidence was downgraded only when the relevant concern was likely to materially influence the interpretation of the network estimate. A lack of statistically significant evidence alone was not considered sufficient to classify the evidence as low or very low confidence

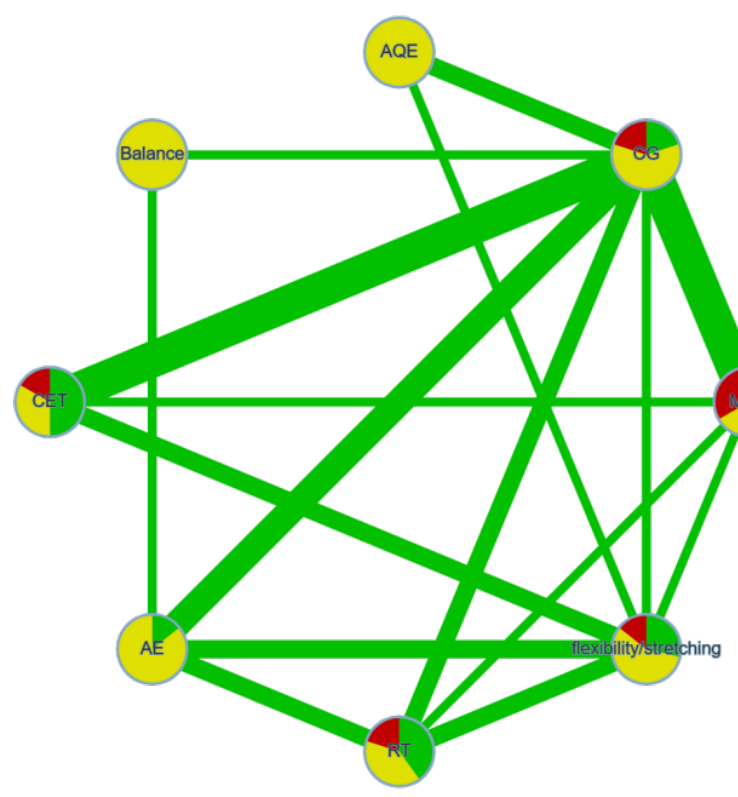

Figure S16. Contribution of study limitations to the network estimates.

The colors in the circles indicate the percentage of low RoB studies (green), studies with some concerns (yellow), high RoB studies (red) for each exercise intervention type. Edge width by the number of studies. Edge color by average RoB. The colors of the lines indicate the summative RoB assessment of each comparison. Low RoB is green, moderate RoB is yellow, high RoB is red. AE: Aerobic Exercise; AQE: Aquatic Exercise; Balance: Balance/Proprioceptive Training; CET: Combined Exercise Training; flexibility/stretching: Flexibility/Stretching Training; MBE: Mind-Body Exercise; RT: Resistance Training; CG: Control Group.

Table S8. Confidence in the evidence using CINeMA for pain intensity outcomes in adults women with fibromyalgia.

Comparison	Within-study bias	Reporting bias	Indirectness	Imprecision	Heterogeneity	Incoherence	Confidence rating	Reason(s) for downgrading
AE:Balance	Some concerns	Low risk	No concerns	Major concerns	No concerns	No concerns	Very low	["Within-study bias","Imprecision"]
**AE:CG**	Some concerns	Low risk	No concerns	No concerns	Major concerns	No concerns	**Very low**	["Within-study bias","Heterogeneity"]
AE:RT	Some concerns	Low risk	No concerns	Major concerns	No concerns	No concerns	Very low	["Within-study bias","Imprecision"]
AE:flexibility/stretching	Some concerns	Low risk	No concerns	No concerns	Major concerns	No concerns	Very low	["Within-study bias","Heterogeneity"]
**AQE:CG**	Some concerns	Low risk	No concerns	Major concerns	No concerns	No concerns	**Very low**	["Within-study bias","Imprecision"]
AQE:flexibility/stretching	Some concerns	Low risk	No concerns	Major concerns	No concerns	No concerns	Very low	["Within-study bias","Imprecision"]
**Balance:CG**	Some concerns	Low risk	No concerns	Major concerns	No concerns	No concerns	**Very low**	["Within-study bias","Imprecision"]
**CET:CG**	Some concerns	Low risk	No concerns	No concerns	Major concerns	No concerns	**Very low**	["Within-study bias","Heterogeneity"]
CET:MBE	Some concerns	Low risk	No concerns	Major concerns	No concerns	No concerns	Very low	["Within-study bias","Imprecision"]
CET:flexibility/stretching	Some concerns	Low risk	No concerns	Major concerns	No concerns	No concerns	Very low	["Within-study bias","Imprecision"]
**MBE:CG**	Some concerns	Low risk	No concerns	No concerns	Some concerns	No concerns	**Low**	["Within-study bias","Heterogeneity"]
**RT:CG**	Some concerns	Low risk	No concerns	No concerns	No concerns	No concerns	**Moderate**	["Within-study bias"]
**Flexibility/stretching:CG**	Some concerns	Low risk	No concerns	Major concerns	No concerns	No concerns	**Very low**	["Within-study bias","Imprecision"]
MBE:RT	Some concerns	Low risk	No concerns	Major concerns	No concerns	No concerns	Very low	["Within-study bias","Imprecision"]

Comparison	Within-study bias	Reporting bias	Indirectness	Imprecision	Heterogeneity	Incoherence	Confidence rating	Reason(s) for downgrading
MBE:flexibility/stretching	Some concerns	Low risk	No concerns	No concerns	Major concerns	No concerns	Very low	["Within-study bias","Heterogeneity"]
RT:flexibility/stretching	Some concerns	Low risk	No concerns	No concerns	Major concerns	Major concerns	Very low	["Within-study bias","Heterogeneity","Incoherence"]

Note: AE: Aerobic Exercise; AQE: Aquatic Exercise; Balance: Balance/Proprioceptive Training; CET: Combined Exercise Training; flexibility/stretching: Flexibility/Stretching Training; MBE: Mind-Body Exercise; RT: Resistance Training; CG: Control Group.

References

1. Altan L, Bingöl U, Aykaç M, Koç Z, Yurtkuran M. Investigation of the effects of pool-based exercise on fibromyalgia syndrome. *Rheumatol Int*. 2004;24(5):272-277. doi:10.1007/s00296-003-0371-7

2. Altan L, Korkmaz N, Bingol Ü, Gunay B. Effect of Pilates Training on People With Fibromyalgia Syndrome: A Pilot Study. *Archives of Physical Medicine and Rehabilitation*. 2009;90(12):1983-1988. doi:10.1016/j.apmr.2009.06.021

3. Andrade CP, Zamunér AR, Forti M, Tamburús NY, Silva E. Effects of aquatic training and detraining on women with fibromyalgia: controlled randomized clinical trial. *Eur J Phys Rehabil Med*. 2019;55(1). doi:10.23736/S1973-9087.18.05041-4

4. Assumpção A, Matsutani LA, Yuan SL, et al. Muscle stretching exercises and resistance training in fibromyalgia: which is better? A three-arm randomized controlled trial. *Eur J Phys Rehabil Med*. 2018;54(5). doi:10.23736/S1973-9087.17.04876-6

5. Atan T, Karavelioğlu Y. Effectiveness of High-Intensity Interval Training vs Moderate-Intensity Continuous Training in Patients With Fibromyalgia: A Pilot Randomized Controlled Trial. *Archives of Physical Medicine and Rehabilitation*. 2020;101(11):1865-1876. doi:10.1016/j.apmr.2020.05.022

6. Baptista AS, Villela AL, Jones A, Natour J. Effectiveness of dance in patients with fibromyalgia: a randomised, single-blind, controlled study.

7. Bircan Ç, Karasel SA, Akgün B, El Ö, Alper S. Effects of muscle strengthening versus aerobic exercise program in fibromyalgia. *Rheumatol Int*. 2008;28(6):527-532. doi:10.1007/s00296-007-0484-5

8. Ekici G, Unal E, Akbayrak T, Vardar-Yagli N, Yakut Y, Karabulut E. Effects of active/passive interventions on pain, anxiety, and quality of life in women with fibromyalgia: Randomized controlled pilot trial. *Women & Health*. 2017;57(1):88-107. doi:10.1080/03630242.2016.1153017

9. Fernandes G, Nery M, Meireles SM, Santos R, Natour J, Jennings F. A functional exercise program improves pain and health related quality of life in patients with fibromyalgia: a randomized controlled trial. *Adv Rheumatol*. 2024;64(1):81. doi:10.1186/s42358-024-00422-7

10. Garrido-Ardila EM, González-López-Arza MV, Jiménez-Palomares M, García-Nogales A, Rodríguez-Mansilla J. Effects of Physiotherapy vs. Acupuncture in Quality of Life, Pain, Stiffness, Difficulty to Work and Depression of Women with Fibromyalgia: A Randomized Controlled Trial. *JCM*. 2021;10(17):3765. doi:10.3390/jcm10173765

11. Hernando-Garijo I, Ceballos-Laita L, Mingo-Gómez MT, et al. Immediate Effects of a Telerehabilitation Program Based on Aerobic Exercise in Women with Fibromyalgia. *IJERPH*. 2021;18(4):2075. doi:10.3390/ijerph18042075

12. Izquierdo-Alventosa R, Inglés M, Cortés-Amador S, Gimeno-Mallench L, Sempere-Rubio N, Serra-Añó P. Effectiveness of High-Frequency Transcranial Magnetic Stimulation and Physical Exercise in Women With Fibromyalgia: A Randomized Controlled Trial. *Physical Therapy*. 2021;101(10):pzab159. doi:10.1093/ptj/pzab159

13. Jones KD, Burckhardt CS, Deodhar AA, Perrin NA, Hanson GC, Bennett RM. A six-month randomized controlled trial of exercise and pyridostigmine in the treatment of fibromyalgia. *Arthritis & Rheumatism*. 2008;58(2):612-622. doi:10.1002/art.23203

14. Kolak E, Ardıç F, Fındıkoğlu G. Effects of different types of exercises on pain, quality of life, depression, and body composition in women with fibromyalgia: A three-arm, parallel-group, randomized trial. *Archives of Rheumatology*. 2022;37(3):444-455. doi:10.46497/ArchRheumatol.2022.9190

15. Ribeiro VGC, Lacerda ACR, Santos JM, et al. Efficacy of Whole-Body Vibration Training on Brain-Derived Neurotrophic Factor, Clinical and Functional Outcomes, and Quality of Life in Women with Fibromyalgia Syndrome: A Randomized Controlled Trial. Schmid M, ed. *Journal of Healthcare Engineering*. 2021;2021:1-9. doi:10.1155/2021/7593802

16. López-Rodríguez MDM, Castro-Sánchez AM, Fernández-Martínez M, Matarán-Peñarrocha GA, Rodríguez-Ferrer ME. Comparación entre biodanza en medio acuático y stretching en la mejora de la calidad de vida y dolor en los pacientes con fibromialgia. *Atención Primaria*. 2012;44(11):641-649. doi:10.1016/j.aprim.2012.03.002

17. Rodríguez-Mansilla J, Mejías-Gil A, Garrido-Ardila EM, Jiménez-Palomares M, Montanero-Fernández J, González-López-Arza MV. Effects of Non-Pharmacological Treatment on Pain, Flexibility, Balance and Quality of Life in Women with Fibromyalgia: A Randomised Clinical Trial. *JCM*. 2021;10(17):3826. doi:10.3390/jcm10173826

18. Sarmento CVM, Moon S, Pfeifer T, et al. The therapeutic efficacy of Qigong exercise on the main symptoms of fibromyalgia: a pilot randomized clinical trial. *Integrative Medicine Research*. 2020;9(4):100416. doi:10.1016/j.imr.2020.100416

19. Valmaña GS, Vidal-Alaball J, Poch PR, et al. Effects of a Physical Exercise Program on Patients Affected with Fibromyalgia.

20. Schachter CL, Busch AJ, Peloso PM, Sheppard MS. Effects of Short Versus Long Bouts of Aerobic Exercise in Sedentary Women With Fibromyalgia: A Randomized Controlled Trial. *Physical Therapy*. 2003;83(4):340-358. doi:10.1093/ptj/83.4.340

21. Silva HJDA, Assunção Júnior JC, De Oliveira FS, et al. Sophrology versus resistance training for treatment of women with fibromyalgia: A randomized controlled trial. *Journal of Bodywork and Movement Therapies*. 2019;23(2):382-389. doi:10.1016/j.jbmt.2018.02.005

22. Valim V, Oliveira L, Suda A, et al. Aerobic Fitness Effects in Fibromyalgia. *The Journal of Rheumatology*.

23. Wang C, Kalish R, Lee Y. A Randomized Trial of Tai Chi for Fibromyalgia. *The new england journal of medicine*. Published online 2010.

24. Yavuz H, Şahinkaya T, Doruk M, et al. FİBROMİYALJİ HASTALARINDA DENGE-PROPRİOSEPSİYON VE AEROBİK EGZERSİZ UYGULAMALARININ FONKSİYONEL DURUM, AĞRI VE DENGE ÜZERİNE ETKİSİ- RANDOMİZE KONTROLLÜ ÇALIŞMA. *jmed*. 2023;86(4):368-375. doi:10.26650/IUITFD.1275105

25. MBNMAdose: Perform a Model-Based Network Meta-Analysis (MBNMA). Accessed August 8, 2026. https://hugaped.github.io/MBNMAdose/articles/runmbnmadose-2.html#model-selection
